# Engineering of Microcage Carbon Nanotube Architectures with Decoupled Multimodal Porosity and Amplified Catalytic Performance

**DOI:** 10.1002/adma.202008307

**Published:** 2021-05-27

**Authors:** Jamie Mannering, Rebecca Stones, Dong Xia, Daniel Sykes, Nicole Hondow, Emmanuel Flahaut, Thomas W. Chamberlain, Rik Brydson, Gareth A. Cairns, Robert Menzel

**Affiliations:** ^1^ School of Chemistry University of Leeds Leeds LS2 9JT UK; ^2^ Henry Moseley X‐Ray Imaging Facility University of Manchester Manchester M13 9PY UK; ^3^ School of Chemical and Process Engineering University of Leeds Leeds LS2 9JT UK; ^4^ CIRIMAT, Université de Toulouse, CNRS, INPT, UPS, UMR CNRS‐UPS‐INP N 5085, Université Toulouse 3 Paul Sabatier Bât. CIRIMAT 118, route de Narbonne Toulouse 31062 France; ^5^ AWE plc Aldermaston Reading Berkshire RG7 4PR UK

**Keywords:** carbon nanotube aerogels, catalysis, emulsion templating, nanoparticle functionalization

## Abstract

New approaches for the engineering of the 3D microstructure, pore modality, and chemical functionality of hierarchically porous nanocarbon assemblies are key to develop the next generation of functional aerogel and membrane materials. Here, interfacially driven assembly of carbon nanotubes (CNT) is exploited to fabricate structurally directed aerogels with highly controlled internal architectures, composed of pseudo‐monolayer, CNT microcages. CNT Pickering emulsions enable engineering at fundamentally different length scales, whereby the microporosity, mesoporosity, and macroporosity are decoupled and individually controlled through CNT type, CNT number density, and process energy, respectively. In addition, metal nanocatalysts (Cu, Pd, and Ru) are embedded within the architectures through an elegant sublimation and shock‐decomposition approach; introducing the first approach that enables through‐volume functionalization of intricate, pre‐designed aerogels without microstructural degradation. Catalytic structure–function relationships are explored in a pharma‐important amidation reaction; providing insights on how the engineered frameworks enhance catalyst activity. A sophisticated array of advanced tomographic, spectroscopic, and microscopic techniques reveal an intricate 3D assembly of CNT building‐blocks and their influence on the functional properties of the enhanced nanocatalysts. These advances set a basis to modulate structure and chemistry of functional aerogel materials independently in a controlled fashion for a variety of applications, including energy conversion and storage, smart electronics, and (electro)catalysis.

## Graphical abstract

Soft‐templating of 1D carbon nanostructures provides next‐generation nanocarbon aerogels with unique microcage internal architectures and independently tuneable micro‐, meso‐, and macroporosities. The intricate aerogel microstructures are chemically modified with metal nanoparticles (Cu, Pd, Ru) through a structure‐preserving sublimation and shock‐decomposition functionalization approach. The subsequent nanoparticle‐decorated aerogels exhibit amplified catalytic performance, as well as solvent‐induced shape‐recovery behavior.

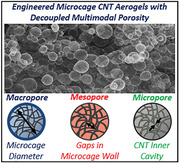

## 1 Introduction

The discovery of atomically thin carbon nanostructures, such as carbon nanotubes (CNTs) and graphene, with an array of impressive and unique physiochemical properties^[^ ^1^, ^2^, ^3^, ^4^ ^]^ has led to intensive research efforts into the development of a diverse range of new nanocarbon‐based applications and technologies, including energy storage,^[^ ^5^ ^]^ water treatment,^[^ ^6^ ^]^ sensing,^[^ ^7^ ^]^ structural composites,^[^ ^8^ ^]^ actuators,^[^ ^9^ ^]^ neural cell growth frameworks,^[^ ^10^ ^]^ and many more.^[^ ^11^ ^]^ Many of these applications require macroscopic nanocarbon material forms with well‐defined structural and chemical characteristics in order to enable integration with existing technological infrastructure. One way in which this can be achieved is through the assembly of individualized nanocarbons into gas‐filled, 3D porous architectures, known as aerogels. Such nanocarbon aerogels exhibit the highly desirable properties of their carbon nanostructure building blocks in a bulk phase while simultaneously reaping the benefits of a hierarchically porous system.^[^ ^12^, ^13^ ^]^ One of the main challenges in nanocarbon aerogel research is to improve control over nanocarbon assembly to produce well‐defined 3D structures capable of optimization for particular applications. Initial work on CNT aerogels resulted in sheet‐like macroscale structures such as vertically aligned nanotube forests and self‐assembled 2D sheets that lack isotropic uniformity, scalability, and simplicity.^[^ ^14^, ^15^, ^16^ ^]^ Early examples of true 3D nanocarbon aerogels (where all three aerogel dimensions are at the same scale) required chemical vapor deposition onto polymeric or metallic substrates and foams.^[^ ^17^, ^18^, ^19^, ^20^ ^]^ These pioneering approaches lay the foundation for numerous solution‐based fabrication methods. There is now a thriving area of research into free‐standing low‐density CNT aerogels fabricated via organogelators capable of gelating the solvent and trapping the nanotubes (e.g., ferrocene‐grafted poly(*p*‐phenyleneethynylene gelation of chloroform)^[^ ^21^ ^]^ and polymer‐assisted assembly (e.g., MWCNT embedded into poly(3,4‐ethylenedioxythiophene)‐poly(styrenesulfonate)).^[^ ^22^ ^]^ These solution‐phase fabrication methods are the first demonstration of true 3D CNT aerogels and are physically characterized as stochastic isotropic architectures.^[^ ^23^, ^24^, ^25^ ^]^ Addressing the lack of detailed control over the internal aerogel microstructure, emulsion‐templating, that is, the directed assembly of nanocarbons at the oil–water droplet interface in Pickering emulsions, offers a highly attractive synthetic strategy. Emulsion‐templating has been successfully exploited to produce GO aerogels with defined, monomodal macroporous closed‐cell internal architectures.^[^ ^26^ ^]^ An interesting feature of emulsion‐templated aerogel synthesis is its scalability, as demonstrated by the fabrication of large scale, free‐standing GO aerogel sheets (1 m^2^), based on significant inhibition of crack formation and propagation due to the disrupted alignment of GO liquid crystals in the presence of a discontinuous oil‐phase.^[^ ^27^ ^]^ Another advantage is the compatibility of emulsion‐templated aerogel synthesis with continuous processing microfluidic techniques to generate superelastic reduced‐GO aerogels.^[^ ^28^ ^]^ The utilization of advanced microfluidic techniques highlights the wide ranging scope of emulsion‐templating synthetic approaches, including their great potential for highly engineered aerogel microstructures. Despite this potential, there are no examples which explore interfacial nanocarbon chemistry for the engineering and modulation of aerogel porosities at different length scales or to achieve this through decoupled, independent processes. 1D carbon allotropes (such as CNTs), which are yet to be utilized, are uniquely advantageous in this regard with the opportunity to yield macroscopic materials with independently controlled hierarchical porosities due to their structural dimensionality.

Beyond this, integration of inorganic nanoparticles (NPs) into aerogels is a rapidly emerging area of interest which enables the tuning of chemical aerogel functionality, important for a wide range of applications, such as energy storage and conversion, sorption, and heterogeneous chemical and electrochemical catalysis.^[^ ^29^, ^30^, ^31^ ^]^ NP‐decorated aerogels provide significant functional benefits over decorated nanocarbon powders, including permanently fixed exfoliation and individualization of the carbon nanostructures, thereby increasing the available active surface of the embedded NPs. There is an extensive set of synthetic techniques developed for the functionalization of unassembled nanocarbon powders.^[^ ^32^, ^33^ ^]^ However, functionalization of 3D nanocarbon architectures is a complex process and only very few approaches have been demonstrated so far. All of these approaches are on stochastic aerogel architectures and utilize wet‐chemical methodologies which typically cause dramatic microstructural changes within the aerogel architecture due to capillary‐force‐driven porosity collapse.^[^ ^34^, ^35^, ^36^ ^]^ There is currently only one reported functionalization approach avoiding wet‐chemical aerogel treatment, based on aerogel functionalization in supercritical CO_2_. However, the extreme pressure conditions and highly turbulent flow environment of supercritical CO_2_ treatments are only suited for the most robust aerogels.^[^ ^37^ ^]^ Here, we propose an alternative, more widely applicable and gentle sublimation–decomposition approach utilizing volatile metal‐complex precursors to enable full, through‐volume, functionalization while negating microstructural changes, even within delicate, pre‐designed aerogel architectures. Sublimation‐based functionalization has proven highly successful for the filling of CNT powders with an assortment of important transition metal NPs.^[^ ^38^, ^39^ ^]^ In context of fine‐chemical catalytic reactions, powders of graphitic nanofibers and single‐wall CNTs (SWCNTs), functionalized through this approach, have been investigated as confinement‐based nanoreactors.^[^ ^40^ ^]^ Developing a sublimation‐based functionalization approach has therefore not only great potential to preserve the microstructure of pre‐designed functional porous materials, but also opens up exciting new opportunities to explore nanoconfinement effects in macroscale aerogel‐based catalyst systems, potentially imparting them with new and unique catalytic functions.

In this study, we utilize 1D carbon allotropes to produce robust, low‐density, double‐wall CNT (DWCNT) and multiwall CNT (MWCNT) aerogels composed of tunable spherical microcages, providing a novel approach toward structurally controllable, uniform CNT aerogels. In addition, we adapt a versatile precursor sublimation–deposition methodology to functionalize the emulsion‐templated aerogels with Cu–CuO, Pd, and Ru metal NPs, introducing a novel, mild aerogel functionalization strategy that enables uniform NP decoration throughout the whole aerogel volume while avoiding irreversible changes in microstructure. The structural and chemical characteristics of the decorated aerogels are characterized at multiple length scales via a range of advanced microscopic, tomographic and spectroscopic materials characterization techniques. To investigate aerogel‐induced impact on NP functionality, Cu–CuO‐decorated DWCNT and MWCNT aerogels are assessed for their catalytic activity in a heterogeneous oxidative amidation reaction and compared with their equivalent unassembled powder analogues.^[^ ^41^ ^]^

## 2 Results and Discussion

**Scheme 1:**
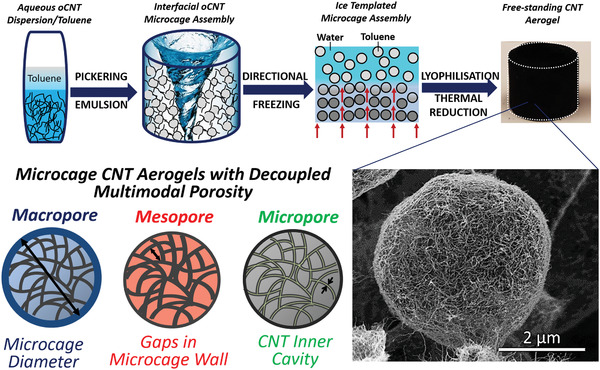
Emulsion templated fabrication (O/W) of uniform free‐standing nanotube aerogels with microcage internal structure. Toluene droplets template the formation of nanotube microcages. The emulsion‐templated nanotubes are locked into position through unidirectional freezing followed by lyophilization and thermal reduction. Crosslinked CNT aerogels exhibit decoupled multimodal porosity controlled by the selection of CNT inner cavity (microporosity), CNT number density (mesoporosity), and droplet size (macroporosity).

**Figure 1:**
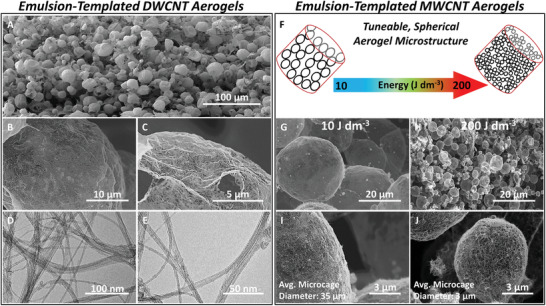
Tunable microcage internal structure of emulsion‐templated DWCNT and MWCNT aerogels. A) Low‐magnification SEM image of the internal microcage structure of a DWCNT aerogel. B,C) Higher‐magnification SEM images of an individual DWCNT microcage revealing an interconnected monolayer of nanotubes. D,E) TEM bright‐field images of the interwoven DWCNT in the microcage walls. F–J) Engineered macroporosity is achieved through template control (F), resulting in: large (35 µm) (G,I) and small (3 µm) (H,J) microcages, depending on emulsification energy input.

As a first nanotube model system, DWCNTs were selected (Figure S1, Supporting Information) as they combine high aspect ratio and high specific surface area (SSA) with a unique two‐wall morphology (allowing for acid‐oxidation of the outer tube while retaining the unique physical properties of the inner tube). Specifically, acid‐oxidized DWCNTs (oDWCNTs) were integrated into an interfacially driven emulsion‐templating aerogel fabrication process (**Scheme** **1**). To this end, the hydrophilic oDWCNT building blocks (Figure S1, Supporting Information) are dispersed and individualized within an additive‐containing aqueous solution at a CNT concentration of around 1.5 mg cm^−3^. At this concentration, a large degree of nanotube de‐bundeling can be achieved without compromising through‐volume nanotube percolation, required for successful formation of robust 3D CNT aerogel networks. Emulsification of the aqueous oDWCNT dispersion with an oil‐phase (toluene, 25 vol%) under pH‐controlled, acidic conditions leads to the formation of nanotube‐stabilized Pickering emulsions. In essence, the spherical oil droplets in these emulsions are coated by the nanotubes and thereby act as highly effective templates for the formation of micrometer‐sized nanotube microcages within the final aerogels (**Figure** **1**).^[^ ^42^, ^43^ ^]^ Careful acidification of the aqueous phase is crucial to drive successful oDWCNT assembly at the oil–water droplet interface via surface group protonation and the resulting slow increase in nanotube hydrophobicity at low pH. The additives (poly(vinyl alcohol) and sucrose) further increase emulsion stability through sites of hydrogen bonding between the polymers and nanotube surface groups; slowing the process of phase separation (Figure S2, Supporting Information).^[^ ^44^ ^]^ The successful formation of CNT aerogels without shrinkage requires a delicate balance of organic additives (OAs) to CNTs at a ratio of 1:2 OA:CNT (Figure S3, Supporting Information). Further, the emulsion‐templated structure is locked in place through unidirectional freezing in custom‐made molds. Growing ice‐crystals from the base upward direct the microcages into vertical columns of alignment to improve macroscale uniformity (Figure S4, Supporting Information). To prevent capillary‐driven collapse, the solvent is removed via freeze drying and the subsequent free‐standing aerogel monolith is reduced under thermal conditions to yield a reduced DWCNT aerogel (Figure S5, Supporting Information) with an ultralow envelope density of around 1.3 mg cm^−3^ (as a comparison the density of air is around 1.2 mg cm^−3^).

Crosslinking of the 3D CNT network is based on substantially increased van der Waals interactions between the reduced DWCNTs due to markedly increased graphitic surface crystallinity after thermal reduction (Figure S2, Supporting Information).^[^ ^45^ ^]^ X‐ray photoelectron spectroscopy (XPS) reveals oxygen functionality (hydroxyl and carboxyl) prior to reduction with a substantial loss in oxygen after thermal reduction (≈1 at% residual oxygen) and marked increase in sp^2^ carbon concentration, Figure S6 and Table S1, Supporting Information).^[^ ^46^, ^47^ ^]^ Subsequent Raman analysis (Figure S7, Supporting Information) confirms a significant increase in graphiticity of the aerogel sample, as indicated by substantial increases in *I*_G_/*I*_D_ intensity ratio and sp^2^ cluster size upon thermal treatment (Table S2, Supporting Information). While the vast majority of the polymer additives are removed during thermal reduction (>95%), some minor residues remain in graphitized form (Figure S7C, Supporting Information) and act as additional covalent crosslinking agents. The resulting stability and robustness of our ultralight nanotube aerogels is highlighted by their ability to resist shrinkage during high‐temperature reduction. In contrast, graphene‐based aerogels, produced at the same low nanocarbon densities and under the exact same fabrication conditions, significantly shrink and their microcellular internal architecture completely collapses upon thermal reduction (Figure S8, Supporting Information), indicating the need for different conditions when fabricating microspherical aerogel architectures from 2D rGO building blocks.^[^ ^26^, ^48^ ^]^ The excellent resistance to microstructural changes highlights the carbon nanotubes as ideal 3D network formers within aerogel fabrication.^[^ ^18^ ^]^ The excellent interconnectivity of 3D nanotube networks also gives rise to reversible mechanical compressibility (Figure S5, Supporting Information) and good through‐volume electrical conductivity in the DWCNT aerogels (0.28 S m^−1^); properties not accessible in other porous materials, such as zeolites or MOFs. Emulsion‐templated aerogel fabrication enables the creation of macroscopic nanotube materials with high surface areas and well‐controlled, hierarchical porosity (**Table** **1**; Figures S9,S10 and Table S3, Supporting Information). During fabrication, the nanotubes remain well‐exfoliated and assemble around the oil droplets in a pseudo‐monolayer fashion, leading to the formation of DWCNT aerogels with a high SSA (785 m^2^ g^−1^), approaching the theoretical limit of fully individualized DWCNTs (800 m^2^ g^−1^ at the same diameter).^[^ ^2^ ^]^ In contrast, the SSA of an equivalent (oxidized, then thermally reduced) DWCNT powder material is only 329 m^2 ^g^−1^, highlighting the strength of emulsion‐templated nanotube aerogels to access the excellent properties of their nanoscale building blocks in the bulk phase.

**Table 1:** Structural characteristics of emulsion templated DWCNT and MWCNT aerogels. Employing DWCNTs with much smaller diameters result in high surface area aerogels with extensive graphiticity. Alternatively, employing MWCNTs lead to aerogels with significantly higher mesopore volume

	Macroscopic properties			Porosity			Graphiticity	
	ρ [mg cm^−3^]	SSA [m^2^ g^−1^]	σ [S m^−1^]	Microcage size [μm]	*V*_Meso_ [cm^3^ g^−1^]	*V*_Micro_ [cm^3^ g^−1^]	*I*_G_/*I*_D_	sp^2^ [nm]
DWCNT aerogel	1.3	785	0.28	30	1.38	0.066	8.36	37
MWCNT aerogel	1.5	416	0.72	35	1.83	0.012	0.79	3

Further, the nanotube aerogels (both DWCNT and later MWCNT) exhibit well‐defined and tunable multimodal porosity on the micro‐, meso‐, and macroporosity length scales as indicated by electron microscopy (Scheme 1; Figure S10, Supporting Information) and nitrogen sorption measurements (Table 1; Figure S9, Supporting Information). The open internal cavities of the nanotubes (i.e., inner nanotube channels) in the microcage walls give rise to very well‐defined microporosity (Figure S1, Supporting Information) which can been tuned through the selection of nanotube building block (e.g., inner DWCNT diameter ≈ 1.5 nm vs inner MWCNT diameter 4 nm, Figure S10, Supporting Information).^[^ ^49^ ^]^ The interstitial spaces between the nanotubes in the mesh‐like microcage walls (Figure 1B,C) generate a substantial degree of mesoporosity (tunable through the selection of CNT type (10–43 nm) and CNT concentration (43–105 µm), Figure S10, Supporting Information). The hollow nanotube microcages create well‐defined macroporosity on the micrometer length scale (≈30 µm), tunable during fabrication through the emulsification energy input (Figure 1F; Figure S10, Supporting Information).

The extension of the emulsion‐templating approach to other 1D nanocarbon allotropes was demonstrated with MWCNTs. Utilizing the same synthetic conditions (Scheme 1), commercially available, carboxylic acid functionalized MWCNTs (Figure S1, Supporting Information) were assembled into MWCNT aerogels with a spherical microcage internal architecture (Figure S11, Supporting Information). Despite having almost identical envelope densities, the DWCNT aerogels and MWCNT aerogels have distinctively different physical and textural characteristics (see differences in SSA, micropore volume (*V*_Micro_), mesopore volume (*V*_Meso_), and electrical conductivity (σ) in Table 1), highlighting simple selection of the CNT building blocks as a straightforward strategy to engineer aerogel microstructure. Elements of these differences are borne out in the microscopy images (Figure 1); a typical internal microcage shows a smooth interconnected network of finely woven DWCNT building blocks (Figure 1B,C). In contrast, the MWCNT aerogels exhibit internal microcages that are less finely woven (Figure 1I,J), resulting in a larger mesopore volume (Table 1). This variation in mesoporosity, at the same carbon density, arises from the differences in diameter and wall thickness of the two nanotube types, resulting in a lower number density and, hence, larger spacing of nanotubes in the MWCNT microcage walls. Selection of CNT type can also be used to tune surface area for a given aerogel envelope density, with the MWCNT aerogel exhibiting a distinctly different, smaller SSA (416 m^2^ g^−1^), compared to the DWCNT aerogel, at the same density (Figure S9, Supporting Information). As for the DWCNT, the SSA of the MWCNT aerogel approaches the theoretical limit of fully individualized MWCNTs of around 500 m^2^ g^−1^. CNT type also impacts on electrical conductivity, with the MWCNT aerogels (0.72 S m^−1^) performing significantly better than DWCNT aerogels (0.28 S m^−1^), reflecting the pronounced metallic character of their MWCNT building blocks.

In terms of aerogel macroporosity, control over the nanotube microcage size in the final aerogels is readily achieved through variation of the shear mixing rate (i.e., energy input, Figure 1F) during the initial emulsification process (Scheme 1, Figure S10, Supporting Information) in order to tune the size of the emulsion droplet templates. Variation of the shear mixing rate in a MWCNT emulsion system was used to reduce internal microcage size by one order of magnitude, from 35 µm at an energy input of 10 J dm^−3^ (Figure 1G,I) down to 3 µm at an energy input of 200 J dm^−3^ (Figure 1H,J). It is worth noting that increasing shear rate during emulsification also results in a much narrower microcage size distribution (Figure S12, Supporting Information). In addition to controlling the micro‐, meso‐, and macroporosity through processing parameters (Figure S10, Supporting Information), a fourth parameter (demonstrated here for the first time with emulsion templated CNT aerogels) of controlling the discontinuous toluene volume fraction (demonstrated between 25–50 vol%, Figure S10, Supporting Information) enables engineering of the fundamental architectural design through an inverse transformation from an open‐cell microstructural architecture to a closed‐cell architecture with wide‐ranging implications on the functionality of percolation networks. Controlling these microstructural parameters, which subsequently enable full control over aerogel porosity and percolation, will inevitably lead to changes in mass transport and provides new opportunities to control and optimize aerogel performance in a given application.

**Figure 2:**
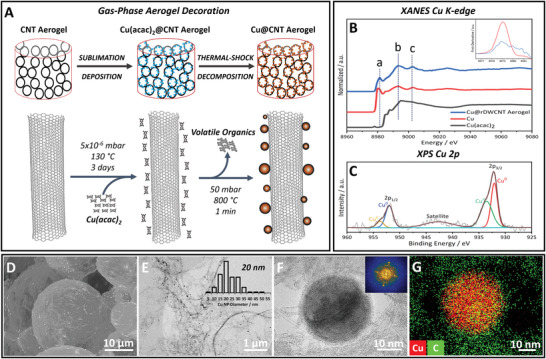
Decoration of CNT aerogels with “naked” metallic Cu–CuO core–shell NPs (Cu@CNT aerogel) through a structure‐preserving, gas‐phase functionalization approach. A) Schematic for the sublimation–deposition of a typical, volatile metal‐complex precursor (Cu(acac)_2_) onto the framework of a nanotube aerogel, followed by thermal‐shock precursor decomposition into Cu–CuO core–shell NPs. B) Synchrotron source X‐ray absorption spectroscopy and C) X‐ray photoelectron spectroscopy of Cu@DWCNT aerogel reveal physiochemical information on the composition of the metallic Cu–CuO NPs. D) SEM backscattered electron mode (BSE). E,F) Bright‐field TEM and G) STEM‐EDX images of Cu@DWCNT aerogel show the structural preservation of aerogel microstructure, Cu–CuO NP distribution. and elemental composition, respectively.

Beyond this, NP decoration is a crucial strategy to widen and tune the chemical functionality of the structurally engineered aerogel materials. Introduction of metal NPs requires a non‐aggressive synthetic methodology, capable of functionalizing the aerogel throughout its entire internal volume without collapsing the carefully designed and intricate microstructure. To achieve this, a stepwise process of sublimation–deposition of readily available transition metal complexes (carrying acetylacetonate or carbonyl ligands) as volatile NP precursors, followed by thermal‐shock decomposition into metal NPs, was employed (**Figure** **2**A). Adapting a well‐established gas‐phase decorative approach, 3D macroscale aerogel structures are subjected to the same sublimation–deposition conditions (Figure 2A) that have proven highly successful for the external and internal functionalization of carbon nanotube and nanofiber powders.^[^ ^38^, ^39^ ^]^ In the context of structurally engineered nanocarbon aerogels, sublimation–deposition of precursor complex compounds have two crucial advantages: i) the NP precursor can readily diffuse through the macroscopic, porous aerogel in the gas‐phase with little mass‐transfer resistance (improving through‐volume decoration uniformity), and ii) precursor deposition can be carried out without exerting any capillary‐forces (so avoiding microstructural aerogel changes). Following precursor sublimation–deposition, a rapid, thermal‐shock‐decomposition process is then applied to efficiently decompose the volatile precursors into metallic NPs (Figure 2A).

As a first model system, this gas‐phase aerogel functionalization approach is demonstrated for the sublimable precursor copper acetylacetonate (Cu(acac)_2_) to decorate emulsion‐templated DWCNT aerogels with Cu–CuO core–shell NPs (a low‐cost and elementally abundant metal with a wide range of applications, especially in chemical and electrochemical catalysis).^[^ ^50^, ^51^, ^52^ ^]^ Stoichiometry of aerogel and precursor are chosen to yield a final Cu–CuO loading of around 8 wt% in the aerogels, a typical degree (between 5–10 wt%) of NP decoration in many inorganic/nanocarbon hybrid powder systems used in catalysis. In the first stage of the aerogel functionalization process, the organometallic precursor is sublimed at moderate temperatures under high‐vacuum conditions and left to diffuse through the aerogel. Precursor deposition onto the aerogel framework is then induced through rapid cooling in an ice‐water bath. Sublimation was carried out here over three days to ensure full precursor utilization; however sublimation times can be readily reduced to hours without any impact on functionalization uniformity (Figure S13, Supporting Information). In fact, this first sublimation stage could be shortened even further (<1 h) through the use of pre‐processed precursor powders with smaller grain sizes (to increase speed of sublimation). In the second stage, Cu–CuO core–shell NP formation is achieved by thermal‐shock heating (800 °C for 1 min) under a low‐pressure nitrogen atmosphere (Figure S14, Supporting Information), inducing rapid precursor decomposition.^[^ ^53^ ^]^ Decomposition was also explored at more conventional heating conditions, including lower temperatures and longer treatment durations; however these decomposition conditions led to relatively large NPs (>70 nm, Figure S15, Supporting Information) or only partial precursor decomposition (Figure S16, Supporting Information). The optimized shock‐decomposition approach, which is applicable to many other metal precursors, yields DWCNT aerogels uniformly embedded with Cu–CuO core–shell NPs (Cu@DWCNT aerogel). The thermal‐shock‐decomposition approach mitigated NP aggregation at longer heating durations (a strategy that has proven highly successful in the synthesis of high‐entropy‐alloy NPs).^[^ ^54^ ^]^ The sublimation/shock‐decomposition approach thereby enables the formation of “naked” Cu–CuO core–shell NPs without any stabilizing capping agents or surfactants; allowing the exposed NP surface to interact directly with the local environment.^[^ ^55^ ^]^

Scanning electron microscopy (SEM) and transmission electron microscopy (TEM) imaging of the final Cu@DWCNT aerogel (sampled from the internal core of the aerogel monolith, Figure 2D–G) confirm the formation of well‐dispersed, unaggregated Cu–CuO NPs and highlight that the gaseous precursor is able to diffuse fully into the aerogel interior. Importantly, SEM imaging collected at lower magnification reveals a fully preserved emulsion‐templated aerogel microstructure after NP decoration (Figure 2D). Bright‐field (BF) TEM and high‐angle annular dark‐field scanning transmission electron microscopy (HAADF‐STEM) reveal that the formed NPs adopt an isotropic, spherical morphology with an average diameter of 20 ± 7 nm (Figure 2E,F; Figure S17, Supporting Information). Interestingly, the DWCNT aerogel induces the formation of 50% smaller NPs with much narrower size distribution, compared to an equivalent DWCNT powder support (Figure S18, Supporting Information). This substantial reduction in NP size (also observed in Cu@MWCNT) and improvement in NP homogeneity likely stems from the much‐improved accessibility of the nanotube's surfaces in the open, porous aerogel network, potentially providing a larger number of more widely spaced NP nucleation sites compared to the heavily aggregated nanotube powders. Increased nucleation and interparticle distances potentially reduce the NP sizes and limit the impact of NP sintering, allowing for aerogel functionalization with smaller NPs relative to the powders. Moreover, high‐resolution TEM imaging and STEM‐EDX mapping (Figure 2G; Figure S17, Supporting Information) confirm that the observed NPs consist of highly crystalline Cu–CuO and are well‐embedded within the carbon nanotube network. The crystallinity of the Cu–CuO NPs is evidenced by the presence of clear lattice spacings in the image and spots in the Fourier‐transformed power spectrum (Figure 2F).

**Figure 3:**
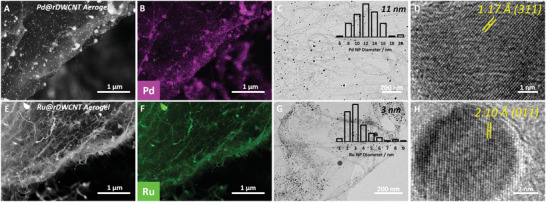
Emulsion‐templated nanotube aerogels decorated with Pd and Ru NPs. A) SEM (BSE mode) and B) SEM‐EDX elemental mapping of Pd@DWCNT aerogel. C) Low‐magnification bright‐field TEM and D) HR‐TEM reveal an average Pd NP size of 11 ± 2 nm in an FCC crystal phase. Similarly, E,F) SEM‐BSE and SEM‐EDX elemental mapping of the Ru@DWCNT aerogel. G,H) Average Ru NP size of 3 ± 2 nm in an HCP crystal phase, determined through TEM.

In order to probe the chemical state of the formed Cu–CuO core–shell NPs, a Cu@DWCNT aerogel was studied via X‐ray absorption near‐edge spectroscopy (XANES), utilizing a high‐energy synchrotron X‐ray source to collect sufficient Cu signal from the challenging ultralow‐density aerogel sample. XANES confirms, on a bulk scale, the presence of metallic Cu^0^ NPs in the decorated aerogels, as indicated by the characteristic Cu K‐edge position (8979 eV) and post‐edge features of the Cu@DWCNT aerogel spectrum (Figure 2B).^[^ ^56^ ^]^ In addition, XANES also confirms the absence of Cu^II^ precursor species (as evidenced by the absence of the Cu^II^ characteristic pre‐edge peak at 8978 eV stemming from the Cu(acac)_2_),^[^ ^57^ ^]^ indicating that the decorated aerogels are free of any unconverted precursor and highlighting the efficacy of the thermal‐shock‐decomposition process in generating Cu–CuO NPs. The post K‐edge features (the extended fine structure) appear slightly shifted (Figure 2B, position b and c) for the aerogel‐supported Cu–CuO when compared to an unsupported Cu^0^ reference sample. This shift suggests a slight difference in local chemical environment, likely arising from factors such as degree of surface oxidation or interaction with the DWCNT framework. However, detailed fine structure modelling (beyond the scope of this study) is required to confirm such effects. To corroborate the XANES results, XPS characterization of the Cu@DWCNT aerogel was employed as a complimentary technique, probing the surface, rather than bulk, of the NPs. The deconvoluted high‐resolution XPS Cu 2p spectrum (Figure 2C) indicates that most Cu in the NP surface layer is present as metallic Cu^0^ (main peak positions at 932.6 and 952.4 eV). However, in contrast to XANES, XPS suggests the additional presence of smaller quantities of Cu^II^ species, which is most likely CuO (weak satellite at ≈943 eV and shoulders in the two main XPS peaks).^[^ ^58^ ^]^ These results suggest a biphasic Cu–CuO which is likely to be in a core–shell composition with a large metallic Cu core and a thin CuO shell, formed upon air exposure. In addition, aerogel decoration via precursor sublimation/shock‐decomposition can be easily translated to other nanotube aerogels (e.g., MWCNT aerogels, Figure S19, Supporting Information) and other metal NPs (e.g., Pd and Ru NPs, **Figure** **3**; Figure S20, Supporting Information). For example, emulsion‐templated nanotube aerogels can also be readily decorated with metal NPs of Pd and Ru (Figure 3), when simple sublimable transition metal complexes, such as Pd(F_6_acac)_2_ or Ru_3_(CO)_12_, are used under the same sublimation–deposition conditions, outlined in Figure 2A. Decoration is again highly uniform across the aerogel framework, as evidenced by SEM–EDX mapping (Figure 3B,F). The formed Pd (11 nm) and Ru (3 nm) NPs are highly crystalline and present in their pure metallic form without any oxide shell, as indicated by high‐resolution TEM (Figure 3D,H) and XPS (Figure S21, Supporting Information).

These results highlight the broad applicability of the sublimation/shock‐decomposition decoration approach utilizing a toolbox of simple metallic complexes and its general compatibility with 3D porous structures.

**Figure 4:**
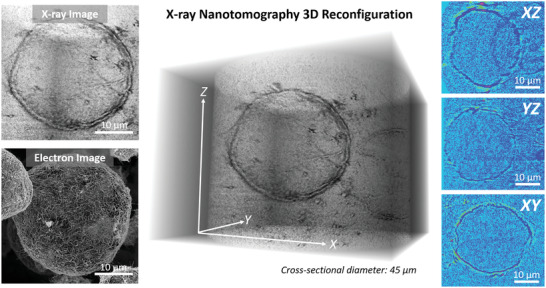
Advanced tomographic characterization on Cu@MWCNT aerogel. Left) Comparison of X‐ray 3D and electron 2D (SEM) images of a typical nanotube microcage. Center) High‐resolution 3D reconstruction of X‐ray nanotomography orthoslices, revealing highly uniform microcages without the presence of large Cu–CuO NPs. Right) Multidimensional analysis of a typical microcage through three different orthogonal planes, reaffirming the highly spherical shape adopted by the nanotubes in emulsion templating.

To image the true 3D aerogel microstructure, X‐ray computed micro‐ and nanotomography was conducted on a Cu–CuO decorated MWCNT aerogel (**Figure** **4**; Figure S22, Supporting Information). X‐ray tomography provides an extremely valuable, non‐destructive tool to probe 3D aerogel morphology and structural uniformity at the micrometer scale without the potentially distorting effects of destructive 3D imaging methodologies, such as FIB‐SEM. The deposited Cu–CuO NPs introduce considerably higher atomic number contrast, thereby acting in effect as contrast agents. The resulting increase in X‐ray attenuation enables faster and more detailed X‐ray tomographic imaging of the emulsion‐templated microstructure (which is very challenging otherwise due to the ultralow‐density of the purely carbon‐based undecorated aerogels). X‐ray microtomography (Figure S22, Supporting Information) shows that the internal CNT microcages are positioned uniformly throughout the aerogel with no observed structural gradients or larger macroscopic voids. Pushing the resolution boundaries in X‐ray imaging, the nanotomography reconstruction (Figure 4) is able to resolve the finer details of a typical nanotube microcage (Figure 4, left panel). The high‐resolution 3D reconstruction (Figure 4, central image) reveals excellent 3D uniformity of the internal microcages, which is further confirmed by the highly spherical color maps of a typical MWCNT microcage in three orthogonal planes (Figure 4, right panel). While the tomography methodologies employed here do not allow for spatial resolution of individual NPs, they do confirm that the sublimation/shock‐decomposition method results in excellent 3D uniformity of Cu–CuO decoration, with the Cu–CuO well‐dispersed around the individual MWCNT microcages.

**Figure 5:**
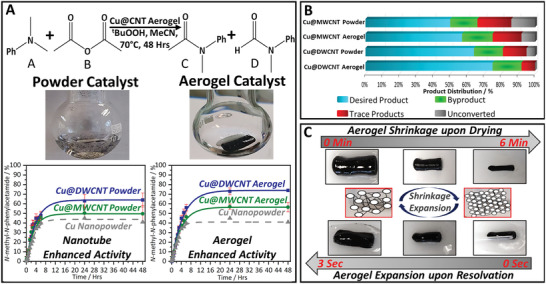
Catalytic performance and solvent‐induced shrinkage‐expansion cycling of emulsion‐templated CNT aerogels decorated with Cu–CuO core–shell NPs. A) Comparison of product formation evolution in an oxidative amidation model reaction, induced by Cu–CuO catalysts supported on different CNT powders and aerogels (Cu loading ≈ 8 wt%), revealing enhanced performance of the Cu–CuO catalyst on the CNT aerogel supports. B) Selectivity based on proportion of the desired product formed in comparison to unreacted starting material and by‐products for different Cu@CNT catalysts, highlighting the excellent catalytic activity of the Cu@DWCNT aerogel. C) Shrinkage–expansion cycling of the Cu@DWCNT aerogel upon drying and addition of acetonitrile (repeated 20 times)—scale bar: 1 cm.

Controlling aerogel 3D microstructure and NP decoration are crucial tools to tailor aerogel materials to specific applications. To probe how structural nanotube assembly impacts on chemical functionality, the emulsion‐templated, Cu–CuO decorated CNT aerogels were studied as catalysts in an oxidative amidation reaction (Scheme S1, Supporting Information). In this context, oxidative amidation (specifically, the reaction of a tertiary amine and an anhydride in the presence of an oxidizing agent)^[^ ^59^ ^]^ provides a particularly robust and repeatable catalytic reaction model, due to the relatively small number of side‐products and the straightforward one step nature of the reaction (reaction scheme **Figure** **5**A) with efficient atom economy. Oxidative amidation reactions are also of great practical importance as amide bond formations are key features in many pharmaceutical compounds (e.g., Lisinopril, Valsartan) and biomolecular structures (e.g., proteins) with 16% of all industrial pharmaceutical reactions involving an amide bond formation stage.^[^ ^60^, ^61^, ^62^ ^]^

In this model reaction system, Cu–CuO decorated CNT aerogels show enhanced product formation yields at equilibrium in comparison to both unsupported NP catalysts as well as Cu@CNT powder analogues (Figure 5A product formation plots, Figure S23, Supporting Information). Moreover, compared to an unsupported, commercial Cu nanopowder catalyst (Figure S25, Supporting Information, Cu@DWCNT aerogel catalyzes oxidative amidation at double the reaction yield and three‐times the turn‐over‐number (TON, see also Table S4 and Figure S26, Supporting Information), indicating markedly improved copper utilization.

The final product yield of the Cu@CNT aerogel catalysts is also enhanced (increase in yield by around 15%) when compared against equivalent Cu@CNT powder analogues (i.e., Cu–CuO NP catalysts supported at the same weight loading on nanotube powders rather than aerogels, Figure 5A). This increase in product yield likely originates from the improved structural features of the aerogel‐based catalyst systems, including larger SSA, higher meso/macroporosity, and smaller NP size compared to the more aggregated powder analogues (Figure S18, Supporting Information). It is worth noting that initial rates for reagent conversion and product formation show less pronounced differences (independent on stirring rate, Figure S24, Supporting Information). Further, reaction yields are limited to about 70%, even for the best performing Cu@DWCNT aerogel catalysts. Post‐catalysis XPS (Figure S27, Supporting Information) suggests that copper oxidation at prolonged reaction times might contribute to limiting the yields. Future reaction optimization (temperature, oxidizing agent) could therefore be utilized to increase final yields even further.

In terms of reaction selectivity, both Cu@CNT aerogel catalysts exhibit a higher selectivity toward the desired amide product compared to their powder analogues—an effect more pronounced for the Cu@DWCNT catalysts (Figure 5B). Full mechanistic understanding of this selectivity improvement in the aerogel‐based catalysts is complex. However, it is plausible to relate selectivity enhancement back to the smaller NP size (potentially leading to changes in type and composition of active sites), higher porosity (potentially impacting mass transport and reaction kinetics) and increased surface accessibility (potentially boosting pre‐concentration and confinement effects) in the aerogel‐based catalysts.^[^ ^63^ ^]^

Interestingly, catalytic aerogel performance can be readily tuned through selection of nanotube building block. The Cu–CuO NP catalysts on the DWCNT aerogel perform considerably better than those on the MWCNT aerogel, as evidenced by a product yield increase of 20% and a doubling of TON (similar increases are reflected in the powder analogues, Table S4, Supporting Information). These differences in catalytic performance likely reflect the markedly different meso‐ and microporosity of the DWCNT and MWCNT aerogel supports (Table 1), impacting on reagent mass transfer and molecular confinement effects. Another potential contributing factor could stem from nanotube‐specific metal–support interactions, originating from the intrinsic electronic and structural differences of the two nanotube types in terms of surface curvature,^[^ ^64^ ^]^ helicity, structural defects,^[^ ^65^ ^]^ and conductivity.

In addition to their tunable catalytic activity (Figure S23, Supporting Information), our metal‐decorated aerogel catalysts also display an interesting and novel dynamic structural behavior on the macroscale (Figure 5C), induced by solvent exposure and removal. Thermal evaporation of the reaction solvent causes the aerogel to shrink significantly and densify. Remarkably, upon addition of fresh solvent/reaction medium the shrunken aerogels fully and rapidly (within seconds) expand back to their original size and shape. This solvent‐induced, dynamic shape‐recovery process is repeatable over 20 cycles without loss of aerogel shape or structural integrity. Shrinkage occurs through the capillary action of evaporation, inducing collapse of the voids within the aerogel. Inversely, a wicking effect is observed on solvent addition, the inner voids balloon which favorably allows shape recovery. This solvent‐induced shape recovery of the aerogels is potentially facilitated by the residual oxygen within the carbon lattice (Figure S6, Supporting Information), improving wettability of the aerogel by the solvent. The flexible network of the high‐aspect‐ratio 1D CNT building blocks crucially enable this dynamic behavior and ensures structural aerogel integrity over repeated shrinkage and expansion cycles. This unusual feature is particularly pronounced in the DWCNT aerogel and to a lesser extent in the MWCNT aerogels. This dynamic structural behavior demonstrates the true multifunctional nature of the nanotube aerogel supports, providing functionality completely inaccessible in unassembled nanocarbon powder materials. The shrinkage/expansion behavior is likely to prove particularly useful for integration of the aerogel catalysts into flow chemistry processes, for example, the aerogel could be easily introduced into a tubular (or more complex) flow reactor architecture and then be expanded upon solvent exposure to fill a predefined volume and shape without any gaps. More generally, we envision future integration of our aerogel catalyst systems into chemical flow processes where they provide a wide range of advantages over catalyst powder systems. The aerogels investigated here show highly controlled porosity (tunable on different length scales) which provides opportunities to control flow properties such as pressure drop, mixing, and residence time, in continuous flow processes. The pronounced (and stable) electrical conductivity of the interconnected CNT aerogel network will also allow for the exploration of electrical current/potential utilization in flow reactions, for example, in context of electrochemical organic flow synthesis, in the future.

## 3 Conclusion

High‐surface‐area aerogel materials with unique microstructural features, based on nanotube microcages, have been successfully produced through an emulsion‐templating methodology. CNT emulsion‐templating allows for tuning of aerogel microstructure across a wide range of length scales through nanotube type selection, nanotube number density, emulsion droplet size and toluene volume fraction. This unique fabrication design provides a multitude of enhanced features such as a SSA approaching the theoretical limit of the selected nanotubes and large, controllable multimodal porosities. Beyond this, the free‐standing aerogels are decorated with metallic NPs through an organometallic precursor sublimation/shock‐decomposition approach. This gas‐phase functionalization approach, demonstrated here in aerogels for the first time, is capable of full diffusion and deposition on the entire aerogel framework without altering its structural design. The rapid, energy efficient and facile shock‐based decomposition produces uncapped, highly crystalline, metallic NPs (Cu–CuO, Pd, and Ru). This sublimation‐based aerogel functionalization approach can be easily extended to include a toolbox of other simple metallic precursors to achieve aerogel decoration with functional mono‐ or multi‐metallic NPs with excellent 3D uniformity. The microstructure‐preserving sublimation/shock‐decomposition methodology can also be readily translated to a wide variety of other structurally engineered macroscopic material forms, such as foams, membranes or woven, fiber‐based fabrics, providing great scope for the development of advanced functional porous materials.

A sophisticated array of advanced characterization techniques spanning all pore size domains (X‐ray nanotomography, SEM, HR‐TEM) enable detailed imaging of the 3D aerogel microstructure while spectroscopic measurements on the bulk and nanoscale (XANES, XPS, STEM‐EDX) enable in‐depth understanding of NP distribution and chemical state. In addition, we explore how the nanotube framework and type of nanotube building block impact on the activity of supported Cu–CuO NPs when they are employed in a pharmaceutical catalytic oxidative amidation reaction. The aerogel supports enhance catalytic performance, with Cu–CuO supported on DWCNTs performing better (higher yields, higher TON, improved selectivity) than the MWCNTs. These findings highlight how simple changes in the nanocarbon building blocks resonate into effects on catalytic performance. The microstructural and chemical tunablity of emulsion‐templated NP@CNT aerogels lay the foundation for future catalyst tailoring for classic catalyzed fine‐chemical synthesis while the aerogels’ electrical responsiveness (enabled by the electrically conducting CNT framework) allow for exciting future exploitation of the engineered materials as well as electrocatalysis for renewable energy vectors and energy storage technologies.

In addition to aerogel‐enhanced catalytic performance, this work provides a blueprint that allows for wider exploitation and integration of microcage CNT aerogels in diverse areas of chemistry and materials science. For example, the capsule‐like internal architectures lend themselves to areas such as drug confinement where microcage wall density and functionality could be tuned to determine drug loading and release rate. The flexibility of the aerogel fabrication process should also prove highly advantageous for future sacrificial templating strategies to produce highly ordered, carbon‐free metal aerogels with engineered microstructures inherited from the sacrificial microcage CNT aerogels. Integration into chemical flow technologies (chemical and electrochemical) is another important future application area envisioned, where the capability to control aerogel structure at different length scales will be highly beneficial, for example by providing unprecedented opportunities to independently tailor through‐flow properties on the mesoscale (enabling control over residence time, reagent mixing, pressure drops, etc.) and molecular confinement on the nanoscale (enabling control of reaction pathways and product selectivity). The discoveries unveiled in this work therefore highlight the potential of these next generation 3D architectures in many well‐established and emerging applications and technologies.

## 4 Experimental Section

### Materials

Catalytic chemical vapor deposition (CCVD) grown, carboxylic‐acid‐functionalized MWCNTs, Cu nanopowder (60–80 nm), *N*,*N*‐dimethylaniline, dodecane, toluene, acetonitrile, Cu(acac)_2_, Pd(F_6_acac)_2_, and Ru_3_(CO)_12_ were purchased from Sigma‐Aldrich. Poly(vinyl alcohol) and sucrose were purchased from Fischer. Acetic anhydride and tert‐butyl hydroperoxide were purchased from Merck. DWCNTs were grown (via CCVD) and acid‐oxidized (3 m HNO_3_, 130 °C, 24 h) using previously reported synthetic methodologies.^[^ ^49^, ^66^ ^]^

### Preparation of CNT Aerogels

Poly(vinyl alcohol) (0.38 mg cm^−3^) and sucrose (0.38 mg cm^−3^) were fully dissolved in water through mild heating. The concentrations of these additives were optimized to maximize mechanical strength while minimizing impact on the nanotube surface‐area (see Figure S3, Supporting Information, and supplementary statement for additive optimization). The acid‐oxidized MWCNTs or DWCNTs (1.5 mg cm^−3^) were added followed by probe‐tip sonication (8 × 10 min, 30% power) with intervals of stirring to produce well‐dispersed aqueous CNTs. HCl (0.1 m, 0.005 vol%) was then added dropwise while under continuous stirring to protonate the surface functional groups, which is required for successful emulsion‐templating. Toluene was added (25 vol%) as the discontinuous phase. The oil/water/CNT mixture was then either vortex mixed (benchtop vortex mixer, Fischerbrand, 10 min at 30 000 RPM) to generate larger microcages or shear mixed (benchtop shear mixer, Silverson L5 fitted with a square hole high shear screen, 10 min, 6000 RPM) to generate small microcages. The emulsion was then unidirectionally frozen in custom‐made molds (15 min), followed by freeze‐drying for 48 h (0.075 mBar, −52 °C, Labconco FreeZone 1). The resulting aerogels were thermally reduced in a tube furnace (100 °C, 5 °C min^−1^, 30 min followed by 1000 °C, 5 °C min^−1^, 2 h) under an H_2_ (5%)/N_2_ atmosphere to facilitate network crosslinking and to restore the graphitic properties of the CNTs.

### Preparation of NPs@CNT Aerogels

DWCNT or MWCNT aerogel and precursor (Cu(acac)_2_, Pd(F_6_acac)_6_, or Ru_3_(CO)_12_) (quantity based on desired weight loading onto aerogel support) were sealed in a Pyrex ampoule (×10^−6^ mbar) on a custom‐made high‐vacuum filling rig. The ampoules were submerged in an oil bath for 3 days at 130 °C followed by rapid cooling through ampoule submersion into ice‐water. The precursor@CNT aerogels were then resealed in a new ampoule under N_2_ (50 mbar) and heated in a muffle furnace (800 °C, 1 min) to form metal decorated CNT aerogels. The same procedure was followed for the CNT powder supports.

### Catalytic Amide Bond Forming Reaction

Acetonitrile (5 mL), dodecane internal standard (0.09 mmol), *N,N*‐dimethylaniline (0.24 mmol), and acetic anhydride (0.29 mmol) were added to an RB flask. A sample (0.2 mL) was taken from the flask and added to CDCl_3_ (0.03% TMS) (0.8 mL) for ^1^H‐NMR analysis (0 h sample). Tertbutyl hydroperoxide (0.19 mmol) was then added followed by the addition of Cu@DWCNT (8 mg), Cu@MWCNT (8 mg), or Cu nanopowder (0.64 mg) catalysts (8 wt% Cu loading). The flask was then placed into an oil bath to initiate the reaction (70 °C, stirring rate 100 RPM under air and standard atmospheric pressure conditions). All further samples were taken with a volume of 0.2 mL and mixed with 0.8 mL of CDCl_3_ (0.03% TMS).

### Characterization

High‐resolution transmission electron microscopy (HR‐TEM) was conducted on a FEI Titan^3^ Themis G2 operating at 300 kV fitted with 4 EDX silicon drift detectors, multiple STEM detectors and a Gatan One‐View CCD camera. EDX spectroscopy and mapping were undertaken using Velox software. A FEI Tecnai TF20 field‐emission gun transmission electron microscope (FEG‐TEM) operating at 200 kV and equipped with an Oxford Instruments 80 mm^2^ X‐Max EDX detector was also used to collect several global view images in order to obtain NP size distributions. Microstructural features were analyzed using a Hitachi SU8230 cold field‐emission scanning electron microscope (FE‐SEM) equipped with an Oxford Instruments 80 mm^2^ X‐Max energy‐dispersive X‐ray (EDX) detector and a photodiode‐backscattered electron (PD‐BSE) detector (2–15 kV). Microscopy images were processed using Digital Micrograph (version 3.30.16.0). X‐ray computed nanotomography was performed using a Zeiss Xradia 810 Ultra with a chromium K alpha source. It was operated at 5.4 keV and Zernike phase contrast was achieved using a phase ring. The sample was centered and a total of 510 equi‐angularly spaced X‐ray projections were taken over a 180° rotation. An exposure time of 90 s/projection was used giving a total acquisition time of 12.75 h (exposure × projections). Volumetric data was reconstructed with voxel size of 129 nm, using a filtered back projection algorithm. X‐ray computed microtomography was performed using a Zeiss Xradia 520 Versa, operated at 60 kV and 5 W. A 10× objective lens was used. A total of 1201 X‐ray projections were collected, over 360° rotation. An exposure time of 15 s/projection was used giving a total acquisition time of 5 h. Volumetric data was reconstructed with a voxel size of 410 nm, using a filtered back projection algorithm. All tomography data processing was conducted using Dragonfly (version 4.1.0.467) software. XANES measurements were taken using the I20 high‐resolution X‐ray emission spectrometer equipped with three Ge(555) analyzer crystals and using the Si(111) monochromator crystal cut.^[^ ^67^ ^]^ Solid samples were mounted between two pieces of Kapton tape and were kept in place by a plastic O‐ring. The spectrometer was calibrated using a Cu foil, measuring the Cu K‐edge. X‐ray photoelectron spectroscopy was undertaken on a Kratos Axis Ultra (Kratos Analytical, UK) spectrometer equipped with a monochromatic Al Kα source (1486.6 eV) and processed using CasaXPS software (version 2.3.22). Surface area and porosity measurements were made on a Micromeritics Tristar 3000 using the Brunauer–Emmett–Teller and the Barrett–Joyner–Halenda methods. Nanocarbon aerogels were degassed on a Micromeritics FlowPrep 060 at 120 °C under a stream of N_2_ for 24 h. Nitrogen sorption isotherm measurements (at a temperature of 77 K) were performed at a relative pressure (*P*/*P*_0_) between 0.05 and 1 with 29 points of measurement in the adsorption stage and 17 points in the desorption stage. Thermogravimetric analysis (TGA) was performed on a Shimadzu TGA‐50 in 50 mL min^−1^ of air flow up to 900 °C (10 °C min^−1^ with a hold at 100 °C for 20 min). Raman spectroscopy was conducted on a Renishaw InVia using a 532 nm laser between 400 and 4000 cm^−1^. H‐NMR was conducted on a Bruker AV3HD 400 MHz spectrometer with a Broadband Observe probe. NMR data was processed using Mnova software (version 14.1.2).

## Conflict of Interest

The authors declare no conflict of interest.

## Supporting information

Supporting Information

## Data Availability

The data that support the findings of this study are available from the corresponding author upon reasonable request.
