# Supplementary material for: Engineering of Microcage Carbon Nanotube Architectures with Decoupled Multimodal Porosity and Amplified Catalytic Performance
Source: Adv Mater. 2021 May 27;33(27):2008307. doi: 10.1002/adma.202008307 (PMC11469132; doi:10.1002/adma.202008307)
Supplement: Supplementary file 1 — Supporting Information [file ADMA-33-2008307-s001.pdf]

© 2021 Wiley-VCH GmbH

ADVANCED MATERIALS

Supporting Information

for *Adv. Mater.,* DOI: 10.1002/adma.202008307

Engineering of Microcage Carbon Nanotube Architectures with Decoupled Multimodal Porosity and Amplified Catalytic Performance

*Jamie Mannering, Rebecca Stones, Dong Xia, Daniel Sykes, Nicole Hondow, Emmanuel Flahaut, Thomas W. Chamberlain, Rik Brydson, Gareth A. Cairns, and Robert Menzel**

Engineering of Microcage Carbon Nanotube Architectures with Decoupled Multimodal Porosity and Amplified Catalytic Performance

Supplementary Information

J. Mannering,[a] R. Stones,[a] D. Xia,[a] D. Sykes,[b] N. Hondow,[c] E. Flahaut,[d] T. W. Chamberlain,[a] R. Brydson,[c] G. Cairns,[e] and R. Menzel*[a]

[a] School of Chemistry, University of Leeds, Leeds, LS2 9JT, United Kingdom

[b] Henry Moseley X-Ray Imaging Facility, University of Manchester, Manchester, M13 9PY, United Kingdom

[c] School of Chemical and Process Engineering, University of Leeds, Leeds, LS2 9JT, United Kingdom

[d]CIRIMAT, Université de Toulouse, CNRS, INPT, UPS, UMR CNRS-UPS-INP N°5085, Université Toulouse 3 Paul Sabatier, Bât. CIRIMAT, 118, route de Narbonne, 31062 Toulouse cedex 9, France CIRIMAT

[e] Atomic Weapons Establishment, Aldermaston, Reading, Berkshire, RG7 4PR, United Kingdom

*Corresponding author: R.Menzel@leeds.ac.uk

Carbon Nanotube Microcage Architectures

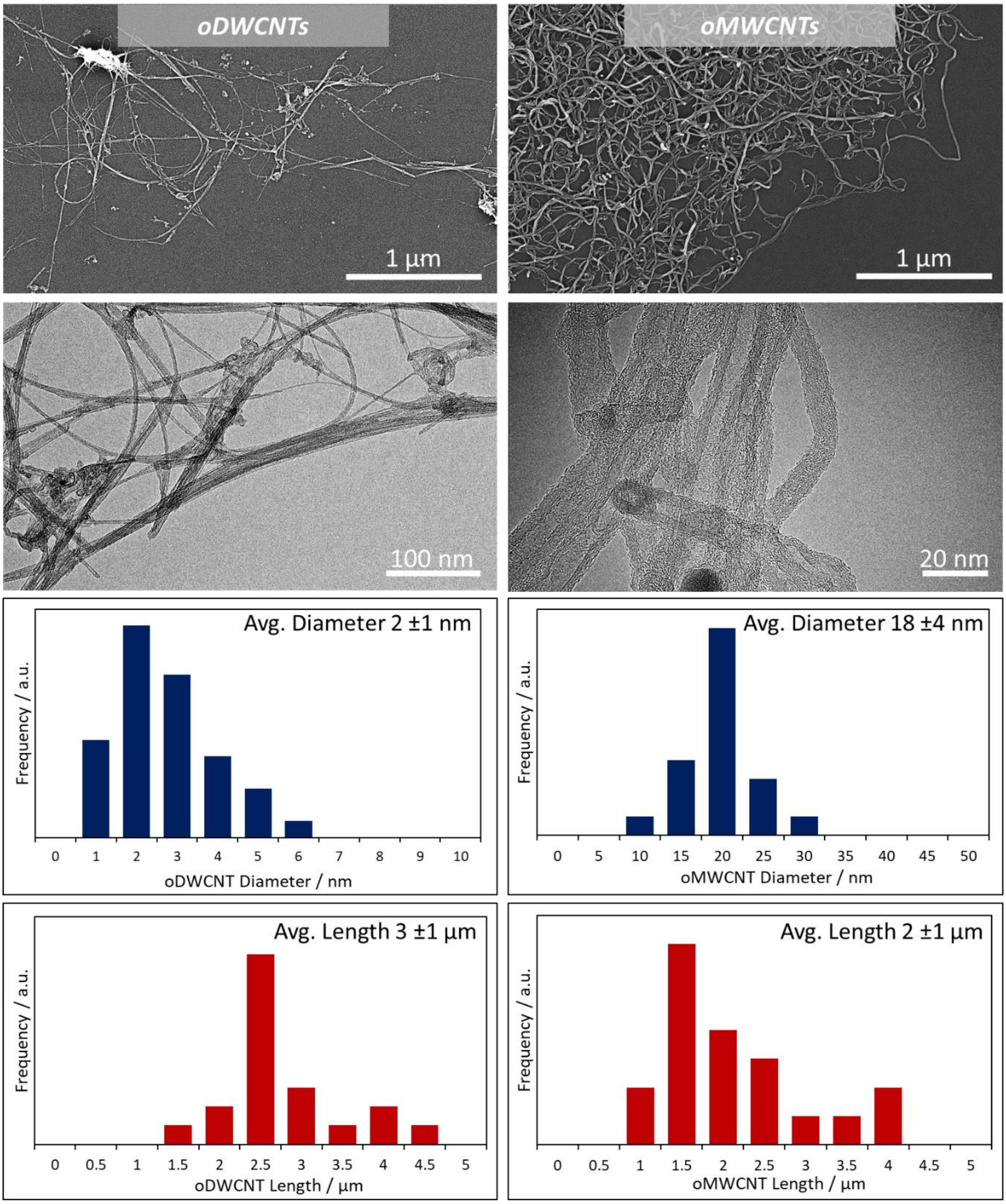

Figure S1. Size and aspect ratio of oDWCNT (left panel) and oMWCNT (right panel) building blocks. Both CNT starting materials exhibit a high aspect ratio; oDWCNTs: 1500:1 and oMWCNTs: 111:1. The SEM, TEM and corresponding size distributions highlight the intrinsic differences between the nanotube building blocks that give rise to differences such as porosity, surface-area, conductivity, NP deposition and catalytic activity in the assembled aerogels.

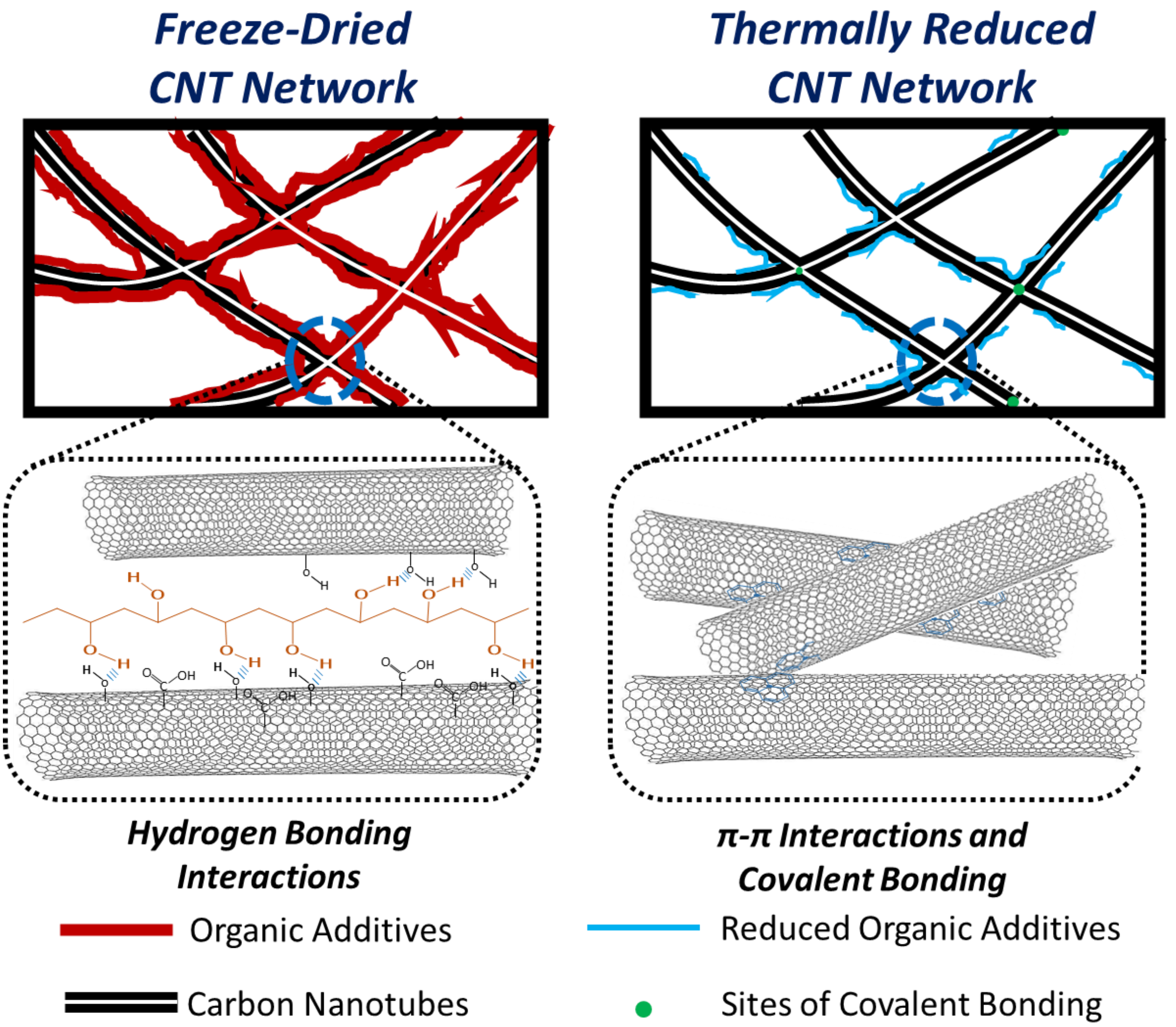

Figure S2. Proposed chemical interactions between nanotubes and organic additives before (left) and after (right) thermal reduction. Prior to reduction, hydrogen bonding dominates the additive-nanotube interactions which are then substituted by aromatic interactions derived from graphitisation of the additives. Additional minor contributions through covalent bonding is likely to occur during high temperature treatment (denoted by green spots).

Carbon Nanotube-Organic Additive Interactions

The specific interactions of PVA with the oxidised nanotubes occurs through hydrogen bonding between the hydroxyl groups of PVA and the polar oxygen-containing functional groups on the nanotube surface, allowing for strong interfacial adhesion. This interaction has been suggested previously and explored through variations in the glass transition temperature of PVA with and without nanotubes as well as shifts in the radial breathing mode (RBM) of the nanotubes.[1] Specifically, PVA is able to hydrogen-bond with the oxidised nanotube starting materials, thereby facilitating CNT exfoliation and dispersion in the aqueous medium. PVA thereby enables a high degree of CNT individualisation, crucial to lower percolation thresholds and to enable efficient 3D network formation in the gelling phase.[2] In addition, the PVA additive is proposed to adopt a second, pivotal role during the emulsion templating stage of our fabrication process. It has been proposed that the PVA polymer chains mediate CNT-CNT interactions in the CNT network, again through hydrogen-bonding. This additional interaction facilitates CNT network assembly and provides mechanical reinforcement to the formed network, which prevents aerogel

shrinkage and deformation during solvent removal (we find that emulsion templated CNT aerogels do not form without the PVA additives upon freeze-drying).

The sucrose additive component also adopts a reinforcing role within the CNT assembly process. Specifically, sucrose functions as a binder improving network stability during the thermal reduction process due to a process similar to liquid-phase sintering. It is believed that the sucrose melts at relatively low temperatures resulting in the formation of high-molecular weight compounds (caramelisation) that reinforce nanotube-nanotube interactions. After subsequent dehydration and decomposition, the sucrose forms graphitised carbonaceous residue (we determine this through Raman spectroscopy on a thermally treated PVA/sucrose mixture, see Figure S7 E), functioning as reinforcement through graphitic crosslinking after thermal reduction.[3]

The concentrations of organic additives (which remained as a 1:1 ratio between PVA and sucrose) were carefully selected to balance the positive impact of additives on the mechanical robustness of the aerogels against the negative impact of additives on aerogel surface area. We find that, at a total additive concentration below 0.75 mg cm^{-3}, the aerogels significantly shrink after freeze-drying and thermal reduction, and show a marked decline in their structural integrity. In contrast, at total additive concentrations above 0.75 mg cm^{-3}, the final aerogels exhibit a significantly reduced surface area (greater than 50% reduction), likely due to relatively large residues remaining within the structure leading to blocked porosity and aggregation/densification leading to reduced surface area. Maximising aerogel surface areas is crucial for NP synthesis (where large surface areas provide a larger number of nucleation sites, resulting in NPs with smaller and more uniform size distributions; see TEM Figure S18), and for catalytic applications (where large surface areas provide a larger number of accessible active sites; see catalytic results). Specifically, we observe a negative impact on the surface area when the total additive concentration was doubled to 1.5 mg cm^{-3}. These findings indicate that the total concentration of 0.75 mg cm^{-3} (0.38 mg cm^{-3} PVA and 0.38 mg cm^{-3} sucrose) is optimal to balance aerogel robustness and surface area whilst maintaining ideal templating conditions.

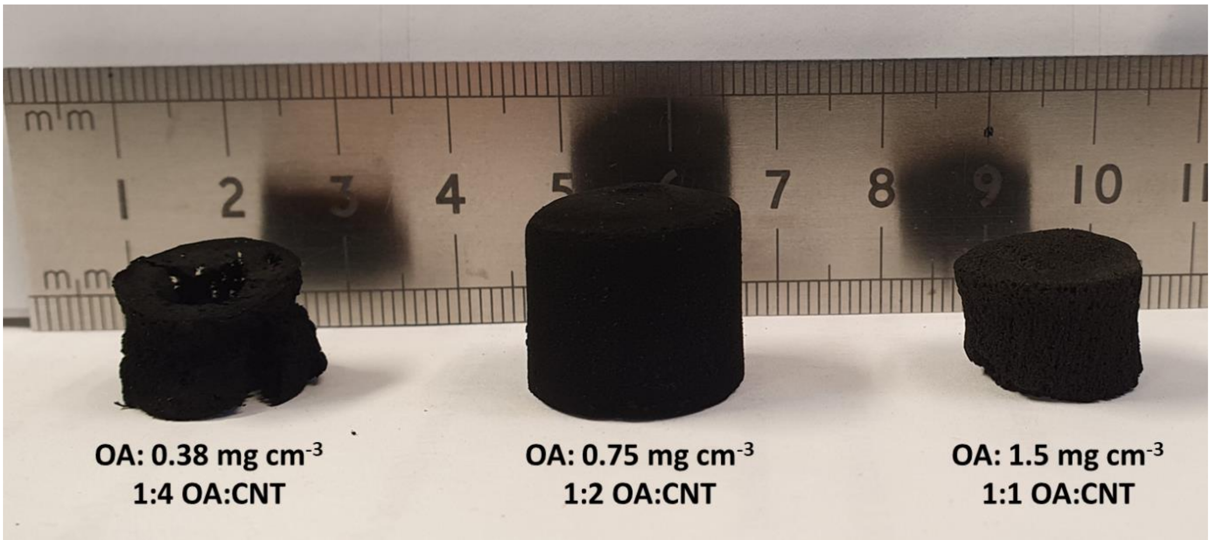

Figure S3. Emulsion templated CNT aerogels under different relative concentrations of organic additives. The optimal concentration of total organic additives was found to be 0.75 mg cm^{-3} with a CNT concentration of 1.5 mg cm^{-3} enabling the formation of free-standing aerogels without shrinkage.

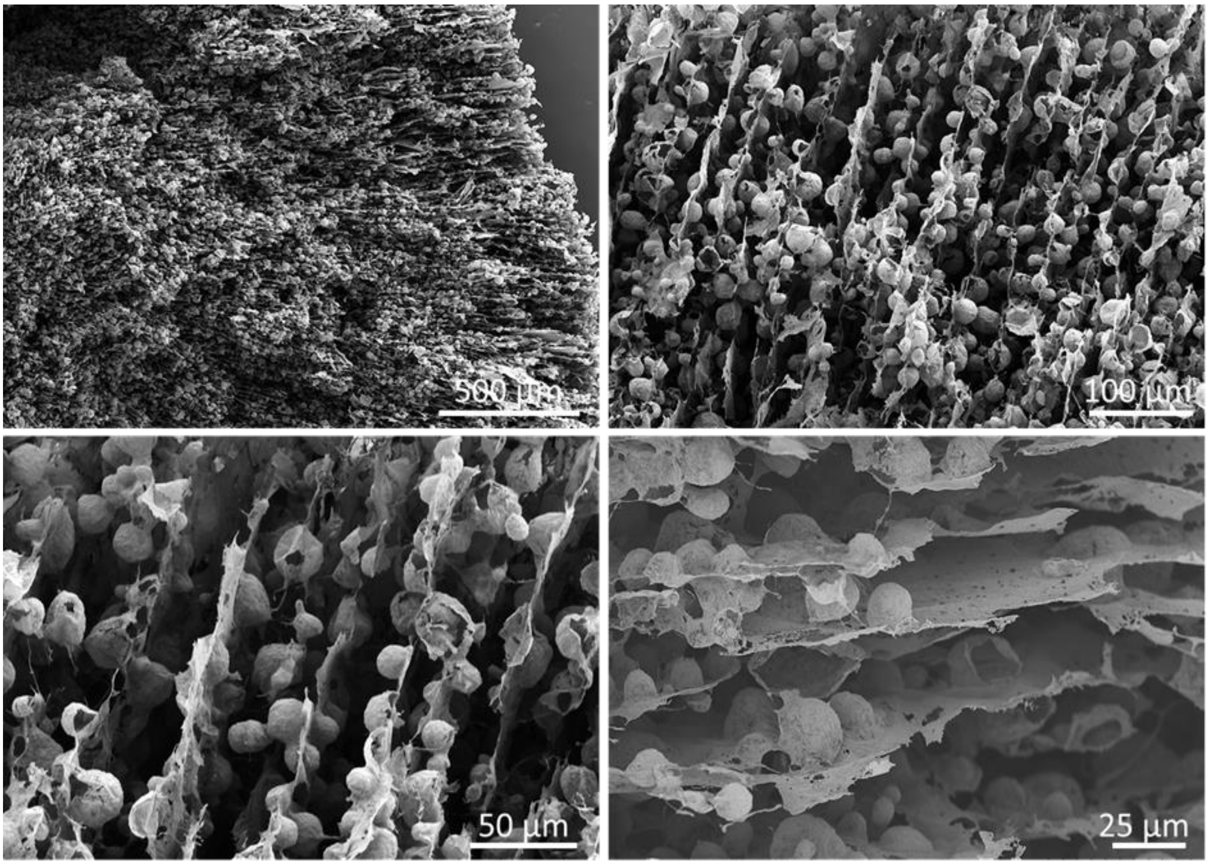

Figure S4. Low-magnification SEM images of DWCNT aerogel, illustrating the alignment of CNT microcages due to the ice growth during unidirectional freezing. In addition to aligning the CNT microcages, these large open channels are ideal for bulk transport of reactants, making them particularly suited for catalytic reactions.

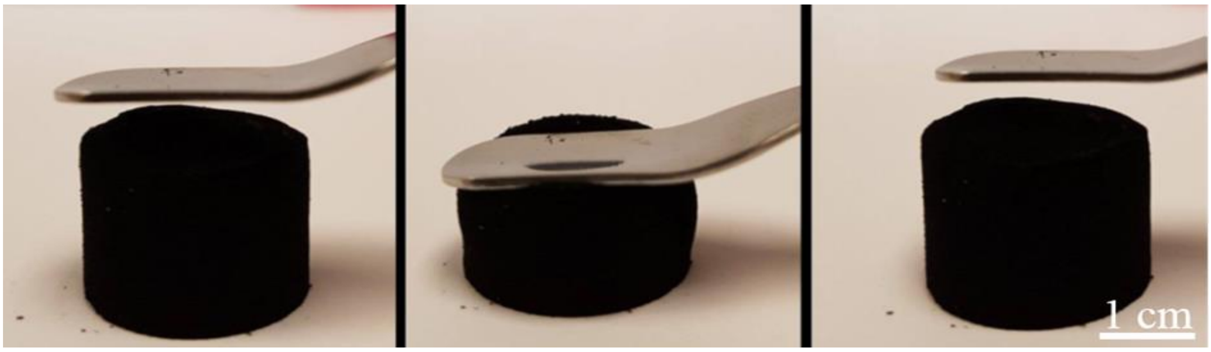

Figure S5. Reversible compression of the emulsion-templated DWCNT aerogel (density 1.3 mg cm^{-3}). Shape recovery demonstrated from 60% aerogel compression. Observationally, the DWCNTs can undergo plastic deformation beyond 60% compression (yield point) whereas the MWCNTs fracture, this is likely related to the higher-aspect ratio of DWCNTs, resulting in greater CNT entanglement and robust mechanical properties.

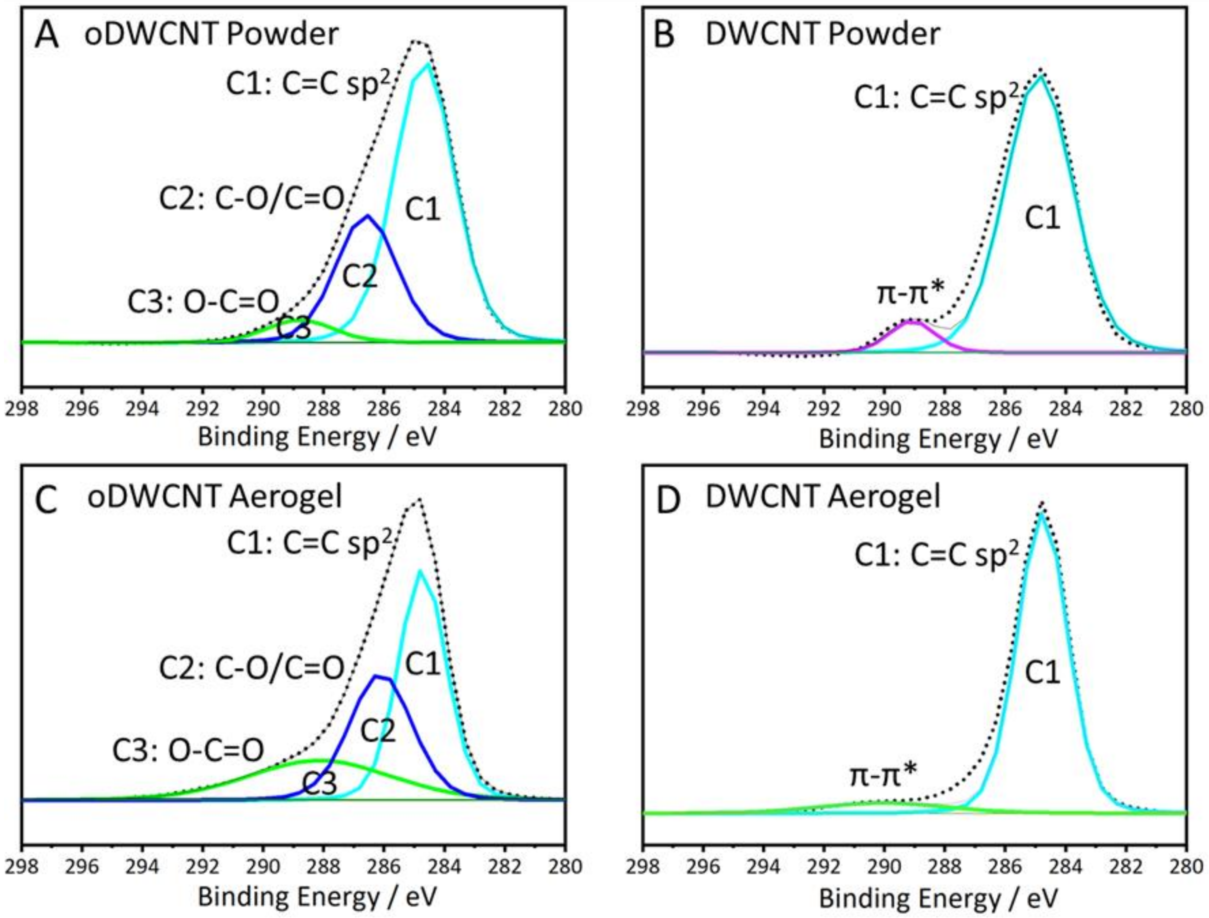

Figure S6. XPS spectra of acid oxidised (A) and thermally reduced (B) DWCNT powder (without organic additives) and acid oxidised (C) and thermally reduced (D) DWCNT aerogel (with added organic additives).

XPS Analysis of Oxygen Functionality

The C 1s spectra for the DWCNT powders (A-B) and aerogels (C-D) before and after thermal reduction was deconvoluted and assigned. Prior to reduction three dominant components are observed (C1-3) corresponding to sp^2 C=C (284.7 eV), C-O/C=O (285.2 eV) and O-C=O (289.1 eV), respectively.[4–8] The XPS data very clearly indicates that there is a substantial increase in sp^2 carbon after thermal reduction (consistent with the pronounced changes in I_D/I_G ratio in the Raman data, ESI Figure S7). We find that the oxygen-containing functional groups (hydroxyl, carbonyl and carboxyl) are removed during thermal treatment. In addition, a small but clear π-π* shake-up peak at increased binding energy is observed for both the powder (289.1 eV) and aerogel (290.5 eV). These observations clearly evidence pronounced graphitisation after thermal reduction treatment, and thereby provide support for our hypothesis that CNT crosslinking in the reduced aerogels is predominantly based on non-covalent van der Waals interactions.

Comparing the C/O atomic ratios of the unreduced aerogel (C/O=80/20) and reduced aerogel (C/O=99/1), it can be seen that oxygen removal is highly effective under the high-temperature (1000 °C) reductive (H_2 in N_2 atmosphere) conditions of the heat treatment. It is also worth noting that about 1 at.% of oxygen remains within the aerogel structures even after this high temperature heat treatment. The nature of these oxygen functionalities cannot be determined (neither from XPS C 1s nor O 1s regions) due to the very low atomic oxygen concentration. However, considering the high treatment temperatures, heterocyclic oxygen functionalities are the most likely candidates (furanes, pyrones etc). While small, these remaining oxygen

functionalities might somewhat modify the surface wettability of the CNTs in the reduced aerogels (compared to extremely hydrophobic as-synthesised CNTs), which might contribute to the pronounced solvent affinity observed during aerogel shrinkage-expansion experiments.

Table S1. XPS quantification of functional groups indicating effective thermal reduction in graphitisation and removal of organics.

	**C1 (C=C sp^2) at.%**	**C2 (C-O/C=O) at.%**	**C3 (O-C=O) at.%**
**oDWCNT Powder**	*64*	*30*	*6*
**DWCNT Powder**	*>99*	*0*	*0*
**oDWCNT Aerogel**	*45*	*32*	*23*
**DWCNT Aerogel**	*>99*	*0*	*0*

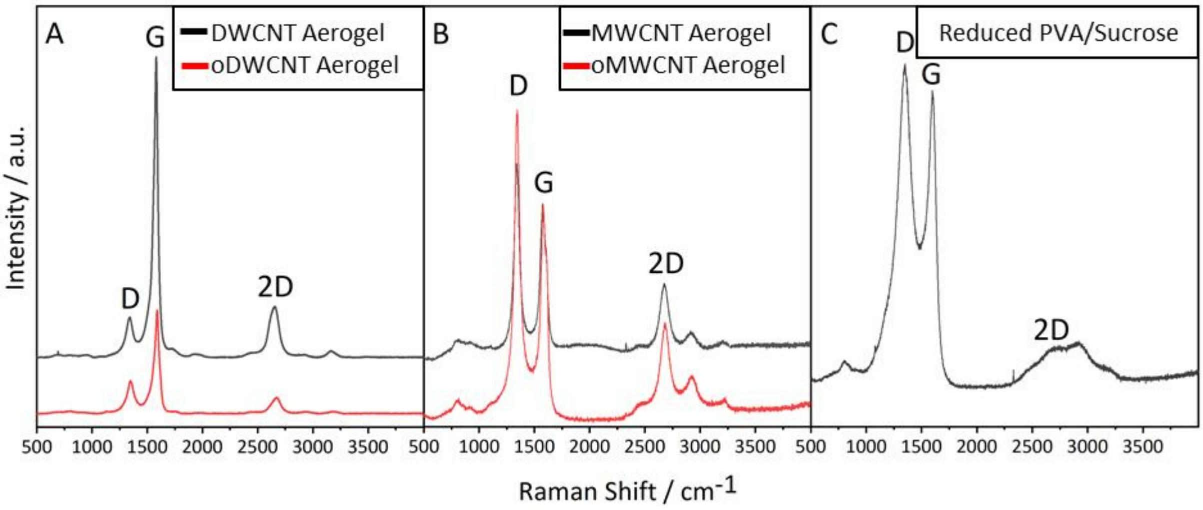

Figure S7. Raman analysis of emulsion-templated nanotube aerogels. (A) Raman spectra of DWCNT aerogel, before and after thermal reduction, showing substantial increase in graphiticity upon thermal treatment, as indicated by a marked increase in the I_G/I_D ratio after thermal reduction. (B) Raman spectra of MWCNT aerogels before and after reduction showing a slight increase in graphiticity post-reduction. (C) Raman spectrum of graphitised additives: thermally-reduced under aerogel fabrication conditions, showing the characteristic D and G peaks of a graphitised material.

Sample	D Peak			G Peak			2D			I_G/I_D
	FWHM (cm^{-1})	I_D	Pos. (cm^{-1})	FWHM (cm^{-1})	I_G	Pos. (cm^{-1})	FWHM (cm^{-1})	I_{2D}	Pos. (cm^{-1})	
**DWCNT Aerogel**	75	19498	1342	44	163037	1578	119	33768	2648	8.33
**oDWCNT Aerogel**	75	16691	1350	54	55113	1586	112	9975	2665	3.33
**MWCNT Aerogel**	56	11021	1341	65	8700	1579	100	4128	2676	0.79

Table S2. | Raman peak values for emulsion-templated nanotube aerogels before and after thermal reduction. Table values were used to calculate intensity ratios and sp^2 cluster size.

Raman Analysis of CNT Aerogels

The characteristic G peak (E_{2g} in-plane phonon mode) is considerably larger after DWCNT aerogel reduction, meaning that there is a significant increase in graphiticity.[9] This is confirmed through a comparison of the D peak (A_{1g} in-plane phonon breathing mode) and G peak in the I_G/I_D ratios, which are found to be 3.33 and 8.33 for oDWCNT and DWCNT aerogels, respectively (Table S2). This is more prominent when compared to the MWCNT aerogel (Table 1) with a ratio of 0.79. At higher wavenumbers, the 2D peak for the DWCNT aerogel, derived from second order zone-boundary phonons, is downshifted by 28 cm^{-1} when compared to MWCNT aerogel; in line with the smaller number of graphene layers in the DWCNT walls.[10] Relating the I_G/I_D ratios to average sp^2 cluster size using the Tuinstra-Koenig relation shows that the sp^2 cluster size is ≈37 nm for the DWCNT aerogel; significantly larger than MWCNT aerogel (3 nm).[11]

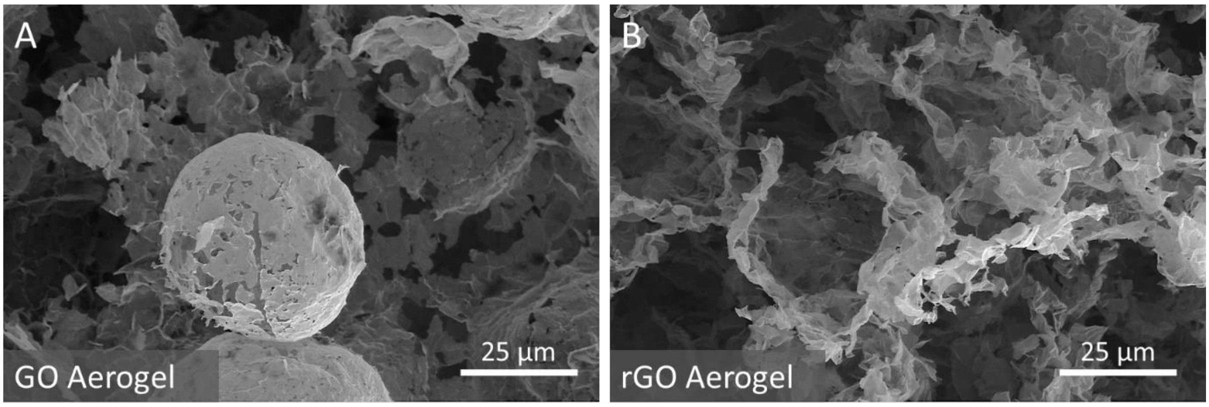

Figure S8. Internal microstructure of an emulsion-templated GO aerogel before (A) and after (B) thermal reduction. SEM images reveal structural collapse of the internal nanocarbon microspheres in emulsion templated GO aerogels after thermal reduction (fabricated using the same synthetic parameters employed in the synthesis of the emulsion templated nanotube aerogels).

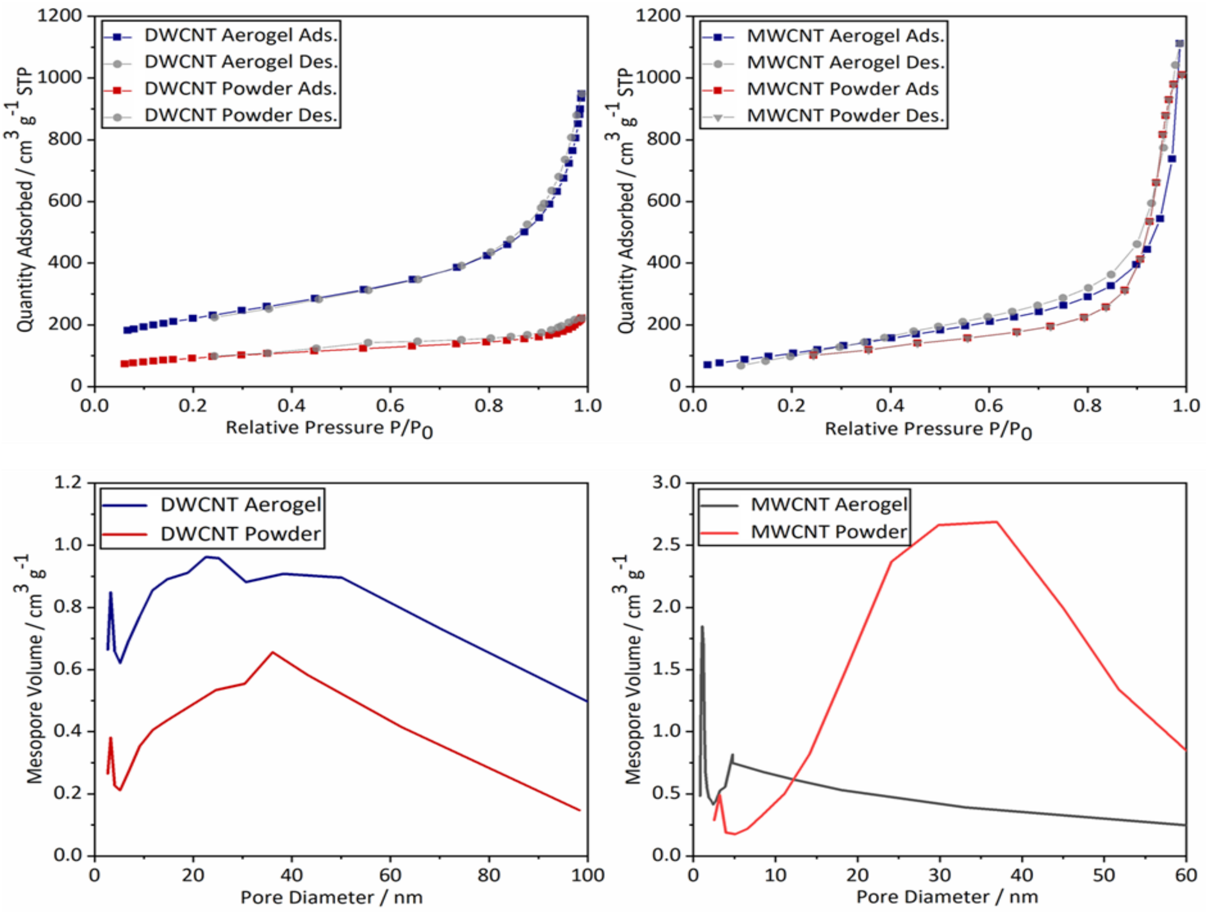

Figure S9. N_2 sorption isotherms and mesopore distributions of emulsion-templated DWCNT (left panel) and MWCNT (right panel) aerogels in comparison to their powder analogues.

N_2 Sorption Analysis of CNT Aerogel Surface Area and Porosity

The N_2 isotherms highlight the considerable increase in specific surface-area (as estimated by BET analysis) of the emulsion-templated aerogels, compared to their powder analogues. The specific surface-area of DWCNT aerogels (785 m^{-2} g^{-1}) more than doubles compared that of the DWCNT powder (329 m^{-2} g^{-1}); a similar observation is made for MWCNT aerogels (416 m^2 g^{-1}) and MWCNT powder (353 m^2 g^{-1}). The high specific surface areas of the aerogels (approaching the theoretical limits for fully individualised DWCNT and MWCNT, respectively) confirm a high degree of nanotube individualisation during wet-chemical assembly, with the final aerogel products, retaining a very high degree of nanotube de-bundling. A high degree of nanotube individualisation in the emulsion-templated aerogels is also indicated by substantial increases in mesoporosity (as estimated by BJH analysis), revealing an up to fivefold increase in mesopore volume in the emulsion templated aerogels compared to their nanotube powder analogues (DWCNTs aerogel: 1.38 cm^3 g^{-1}, powder: 0.28 cm^3 g^{-1} and MWCNTs aerogel: 1.83 cm^3 g^{-1}, powder: 1.53 cm^3 g^{-1}). As mesoporosity arises from the interstitial gaps between nanotubes, the substantially increased meso-porosity of the emulsion-templated aerogels indicates the presence of highly de-bundled nanotubes in the aerogels' internal microcage walls. The DWCNT aerogels, which exhibit a finer microcage mesh, displays a far greater increase in mesopore volume at a lower mean meso-pore diameter.

Table S3. Summary of porosity variation demonstrated through four different synthetic parameters; selection of CNT type (microporosity), CNT concentration (mesoporosity), emulsification energy (macroporosity) and toluene volume fraction (microcage packing efficiency).

Synthetic Parameter	Structural Parameter	Tuneable Size Range
CNT Type	Microporosity	1.5-4 nm
CNT Concentration	Mesoporosity	43-105 nm
Emulsification Energy	Macroporosity	3-35 µm
Toluene Volume Fraction	Packing Efficiency	15-30 µCage/µm^2

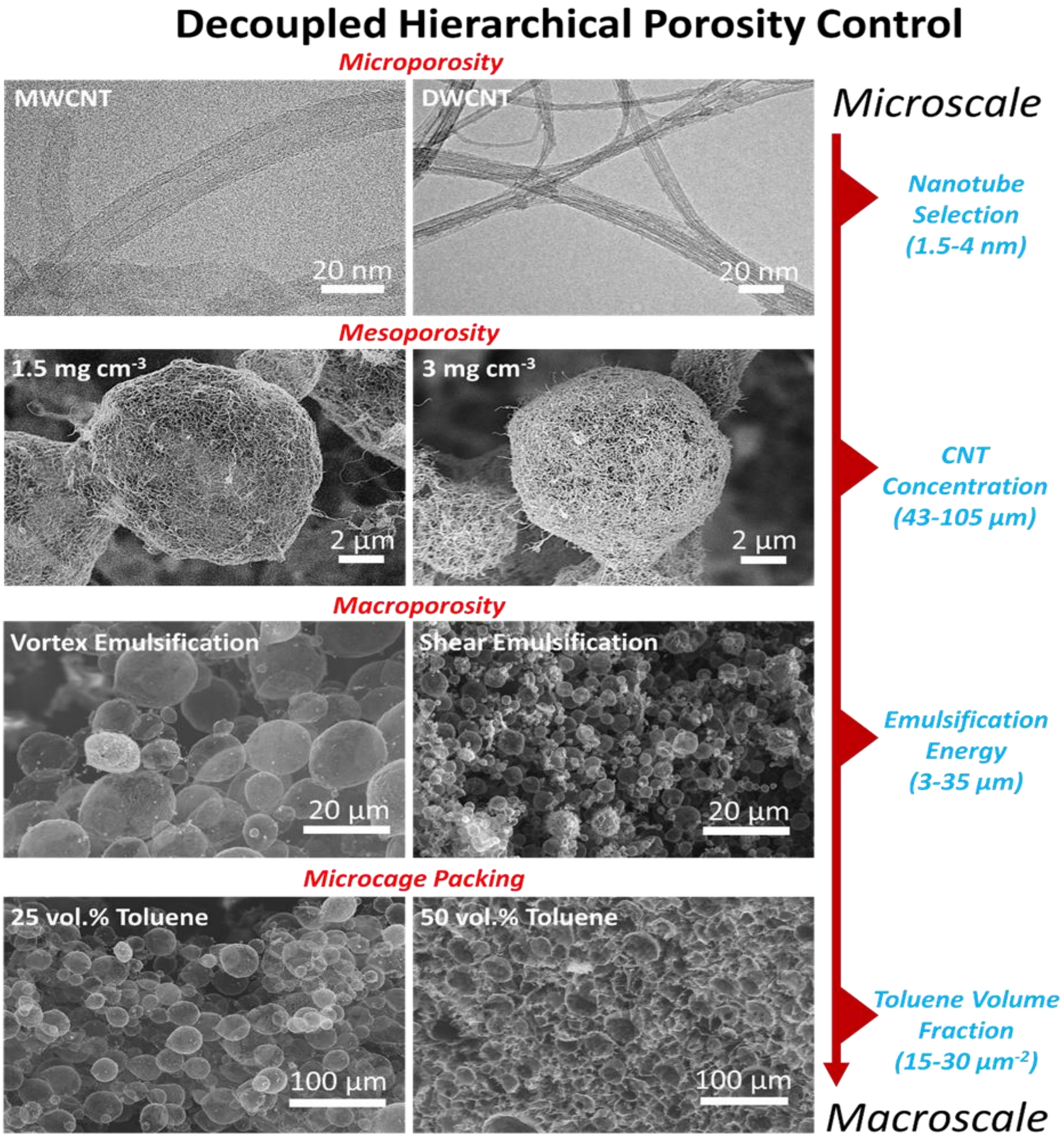

Figure S10. Engineering the aerogel microstructure and porosity through decoupled independent parameter control. Selection of nanotube type, nanotube concentration and emulsification energy enable micro-, meso- and macroporosity tuning whilst toluene volume fraction induces an inverse transformation from open-cell morphology to closed-cell morphology (i.e. enabling 3D percolation control).

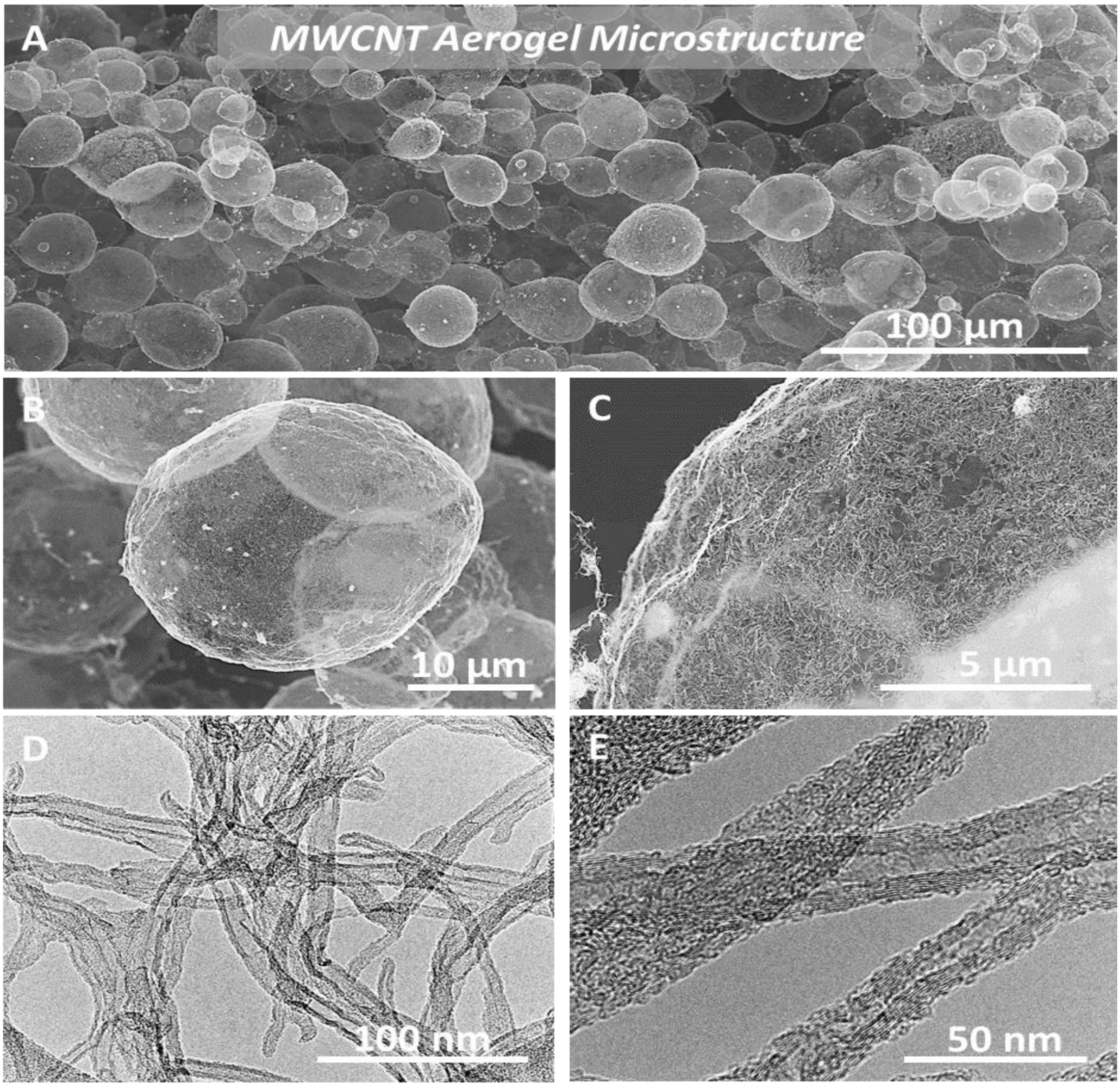

Figure S11. Emulsion-templated MWCNT Aerogel. (A) SEM images of global view distribution of MWCNT microcages revealing their balloon-like microstructure. (B, C) Higher magnification SEM images of a typical microcage revealing an interconnected network of nanotubes. (D, E) TEM images of the individual MWCNTs highlighting the diameter and inner cavities.

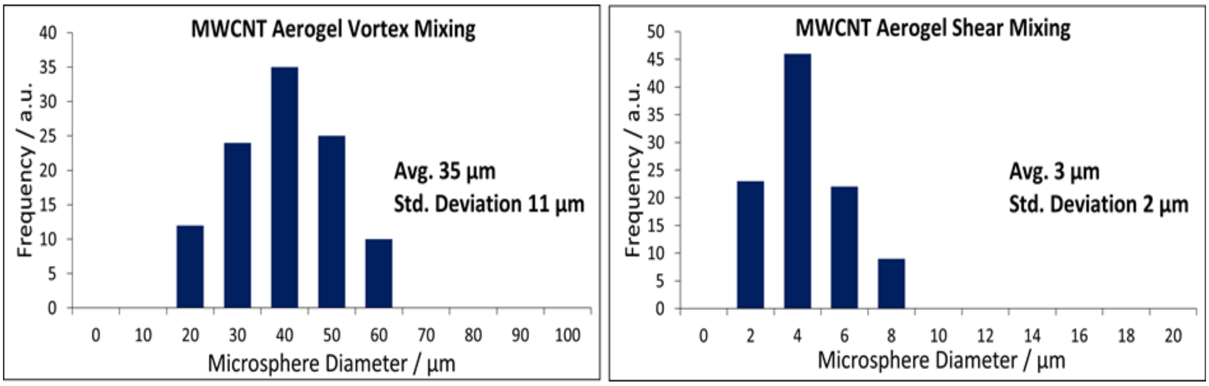

Figure S12. Internal microcage size distributions of emulsion-templated MWCNT aerogels, produced at different emulsification energies. Comparison of the microcage size distribution in low-energy vortex emulsification (energy input 10 J dm^{-3}) and high-energy shear mixing emulsification (energy input 200 J dm^{-3}).

Functionalisation of Carbon Nanotube Aerogels

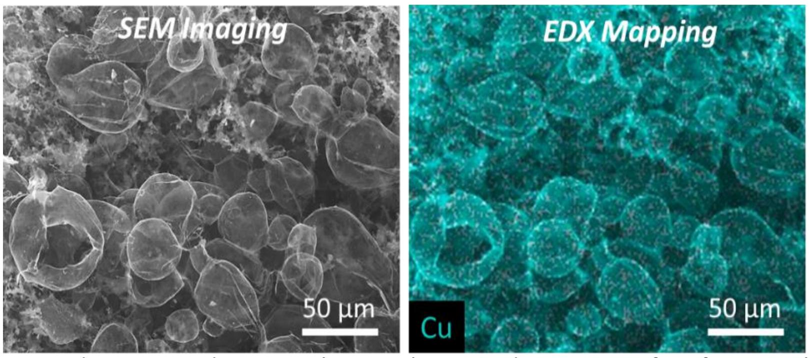

Figure S13. SEM image and corresponding EDX elemental map and spectrum of Cu functionalised CNT aerogel core under a reduced sublimation exposure time (10 hrs).

Sublimation and Shock-Decomposition Optimisation

We find that full sublimation of the Cu(acac)$_2$ precursor powder depends on the grain sizes. Fine precursor powders sublime within several minutes whereas the larger, more compact precursor grains (often distributed amongst the finer powder even with mild grinding), require considerably more time. Under these conditions, at 10 hours the entire sublimation process has occurred which enables through-volume functionalisation. The sublimation process could be shortened considerable to under 1 hr. Strategies to achieve this is additional processing of the Cu(acac)$_2$ powder via ball milling to reduce grain sizes.

Optimization of the thermal decomposition process of Cu(acac)$_2$ during the second stage of functionalisation required screening of several temperature and duration profiles. This work finds rapid-shock decomposition to be the most efficient and effective whilst also being universally applicable to other metal precursors.[12] Adopting lower decomposition temperatures led to significant NP sintering and polydispersity. An example of this was a temperature/time screening process conducted at 300 °C where decreasing decomposition time led to progressively smaller Cu-CuO core-shell NPs (Figure S14). However, below 10 mins only partial Cu(acac)$_2$ decomposition occurred which is observe through NP morphology change and the formation of a non-crystalline carbonaceous shell under e-beam exposure during TEM (Figure S15). These observations were also made at other temperatures such as 500 °C indicating difficulty in achieving the balance of full-decomposition whilst maintaining uniformly distributed and relatively small (<20 nm) spherical NPs.

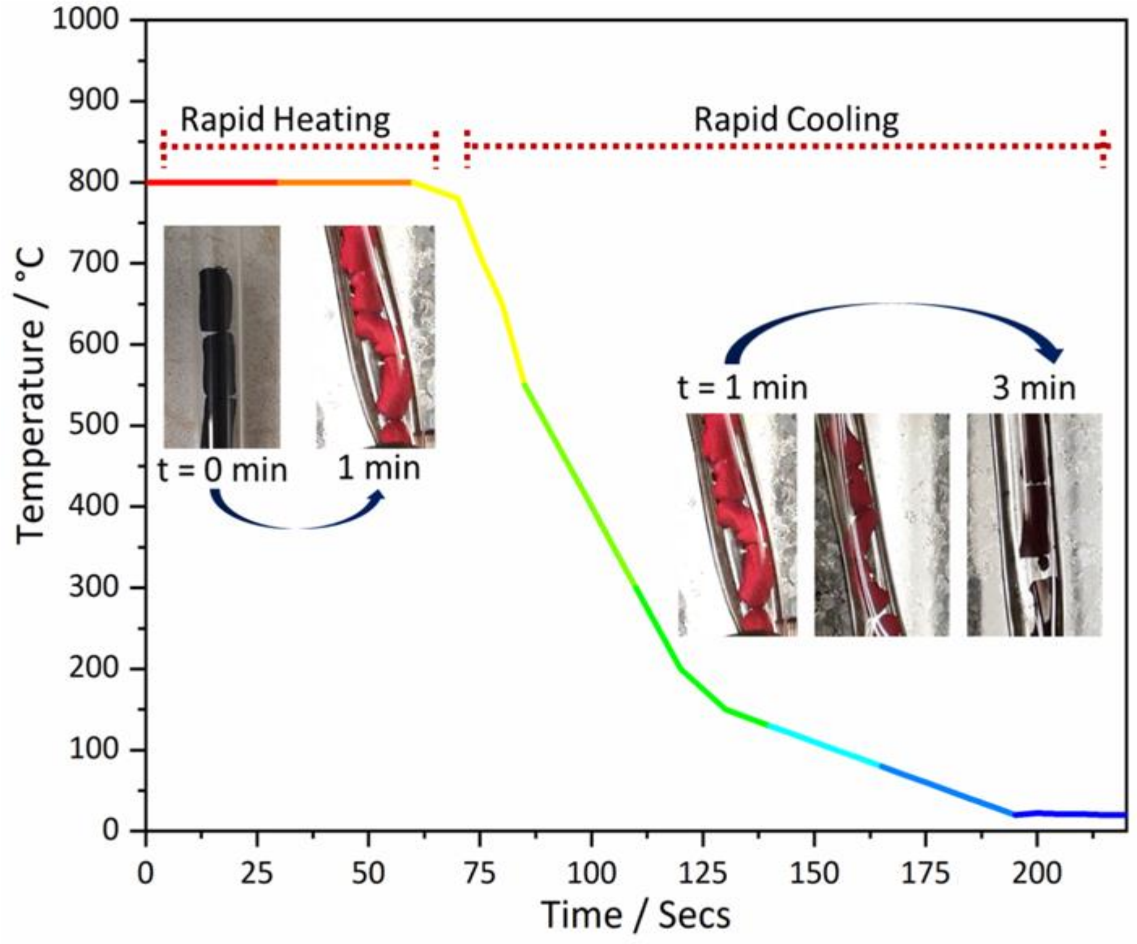

Figure S14. Thermal shock decomposition of metal-organic NP precursor compound. Emulsion-templated nanotube aerogel monoliths decorated with a transition metal compound complex are sealed in ampoules under low-pressure nitrogen conditions. Ampoules are then rapidly heated in a pre-heated furnace to 800 °C for 1 min, followed by fast natural cooling to ambient temperature in less than 3 min. The time-temperature profile for the precursor decomposition process illustrates the rapid heating and cooling kinetics involved, enabled by the excellent thermal stability and thermal conductivity of the CNT building blocks. Aerogel black-body thermal emission of red light at 800 °C and a colour change back to black illustrate the pronounced and rapid temperature changes involved.

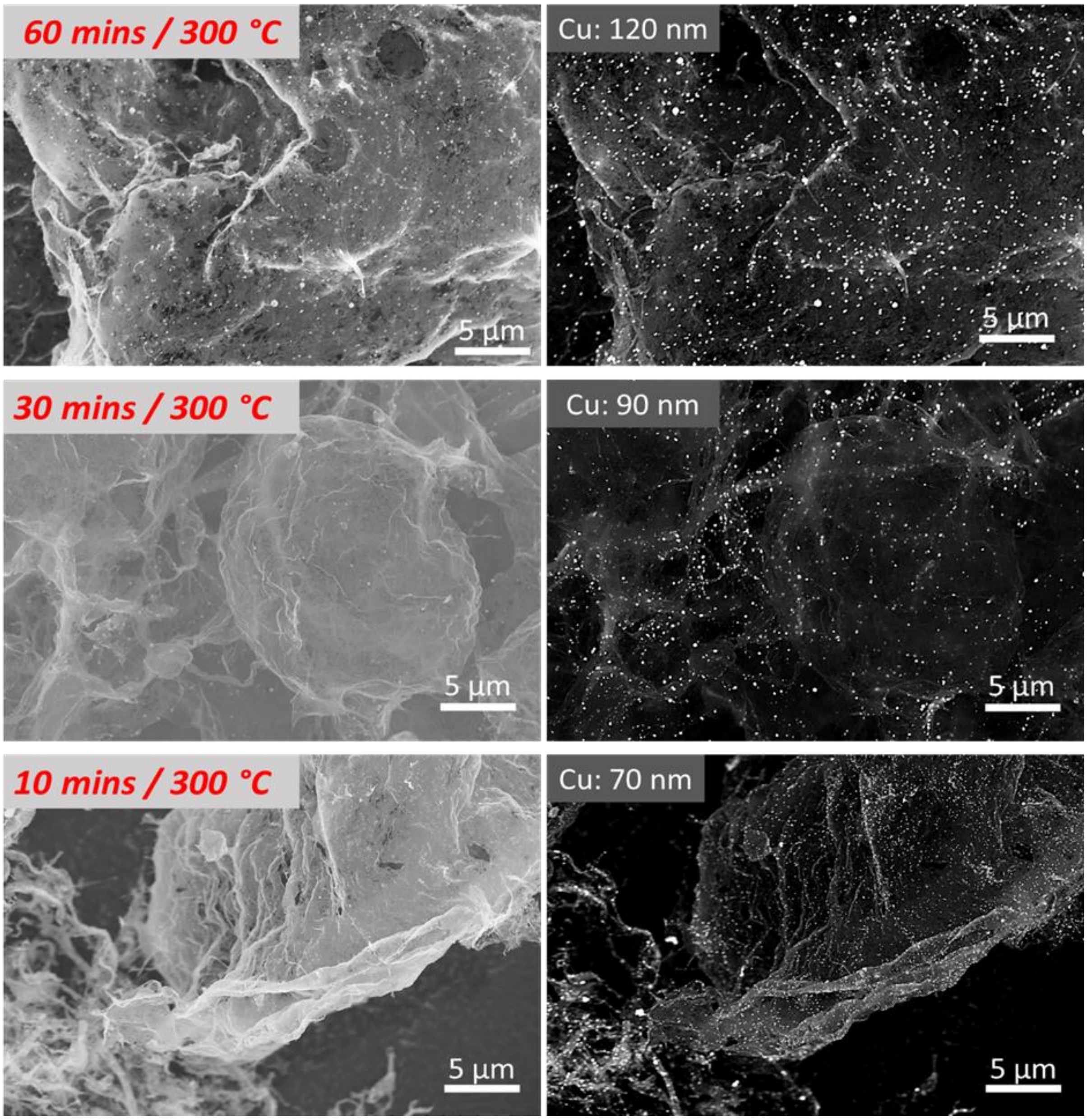

Figure S15. Example of the optimisation screening of decomposition temperature and duration at 300 °C for 60 mins, 30 mins and 10 mins. An alternative rapid shock-decomposition proved to be the most effective in generating uniform, well-distributed Cu-CuO core-shell NPs (<20 nm).

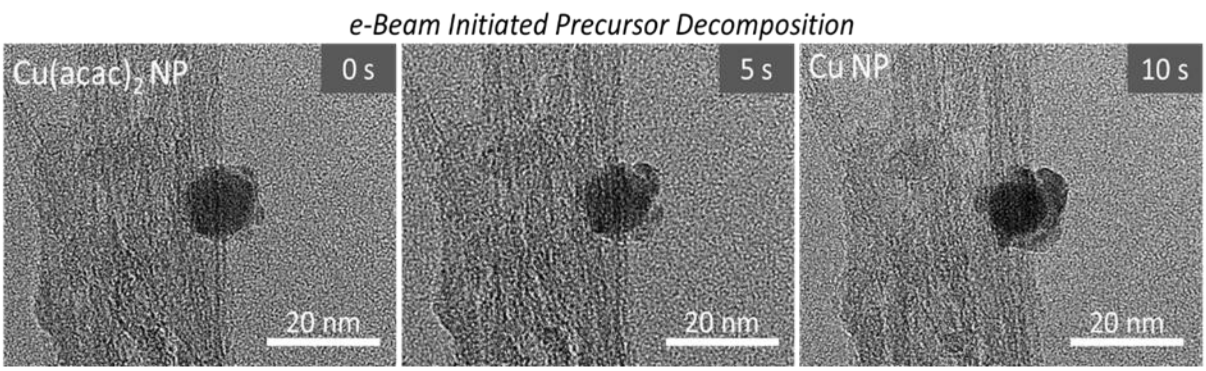

Figure S16. $Cu(acac)_2$ precursor decomposition under e-beam irradiation within the sub-optimised (<10 mins at 300 °C) functionalization conditions.

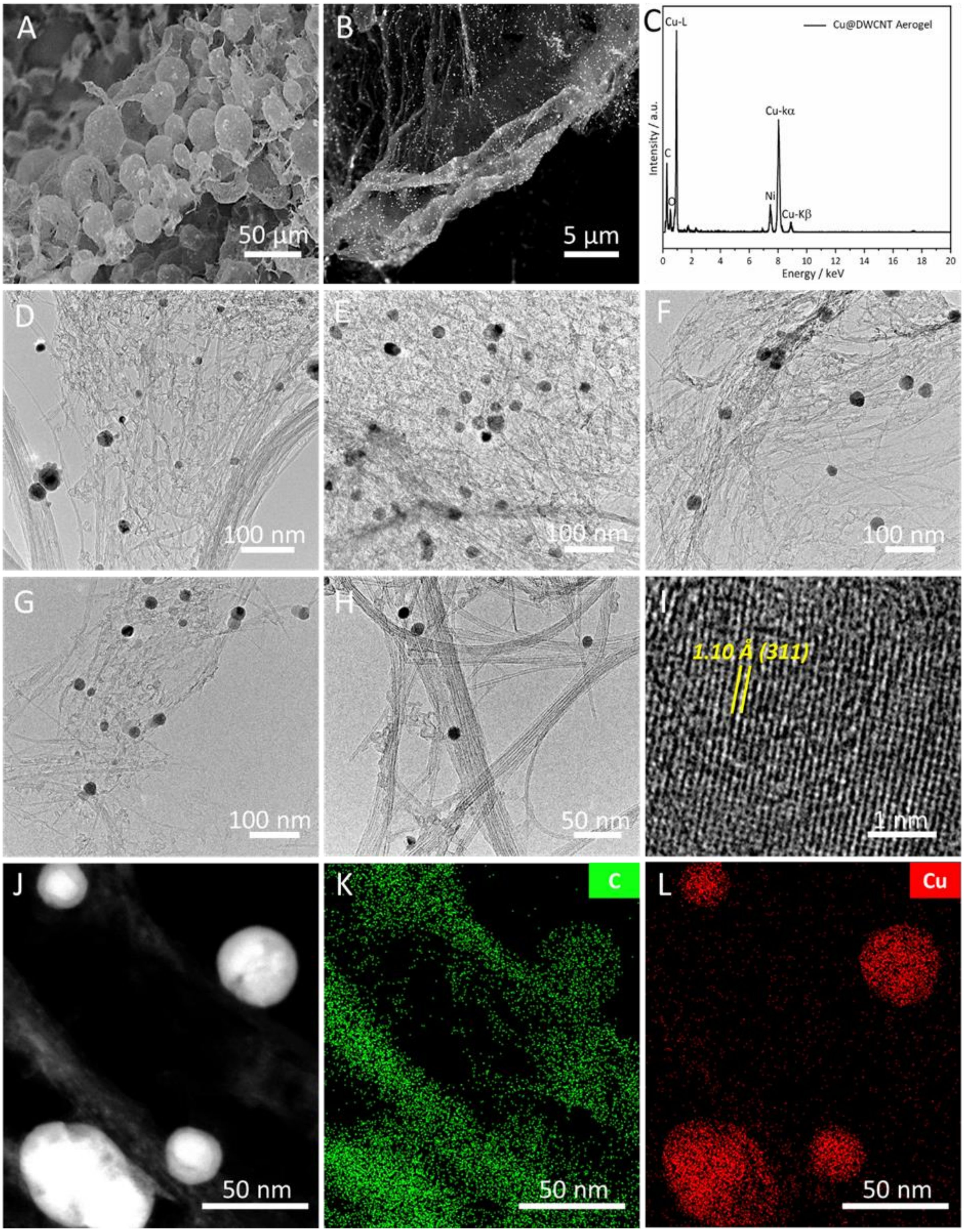

Figure S17. Electron microscopy and spectroscopy of Cu-CuO core-shell NPs supported on DWCNT aerogel. (A,B) SEM-BSE images of the fully preserved aerogel microstructure after NP decoration. (C) TEM-EDX spectrum confirming the presence of copper along with the detection of carbon (carbon nanotubes) and nickel (TEM grid). (D-H) Low-magnification TEM images revealing the distribution, morphology and size of the supported Cu-CuO core-shell NPs. (I) HR-TEM lattice spacing of 1.10 Å indicates the presence of the metallic Cu in its FCC crystal phase (311(FCC Cu) = 1.10 Å). (J-L) STEM-EDX elemental mapping of the Cu@DWCNT aerogel - carbon (green) and Cu (red).

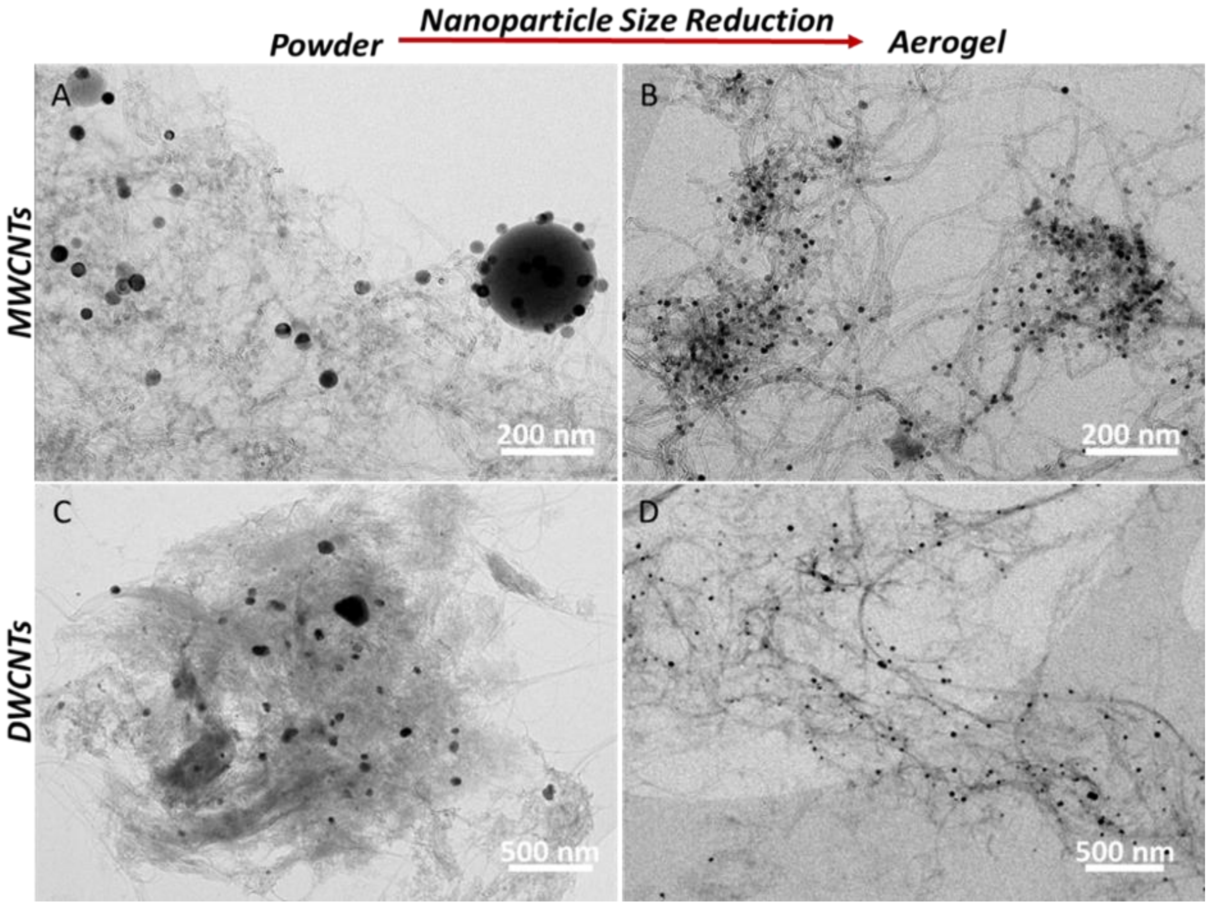

Figure S18. Comparison of Cu-CuO decorated CNT aerogels and Cu-CuO decorated CNT powders. TEM images of Cu-CuO core-shell NP catalysts supported on different CNT materials (Cu weight loading around 8 wt.% for all samples): A) Cu@MWCNT powder, B) Cu@MWCNT Aerogel, C) Cu@DWCNT powder and D) Cu@DWCNT Aerogel. The comparison between powder and aerogel supports reveals a clear reduction in particle size and substantial increase in homogeneity for the aerogel-supported Cu-CuO core-shell NPs.

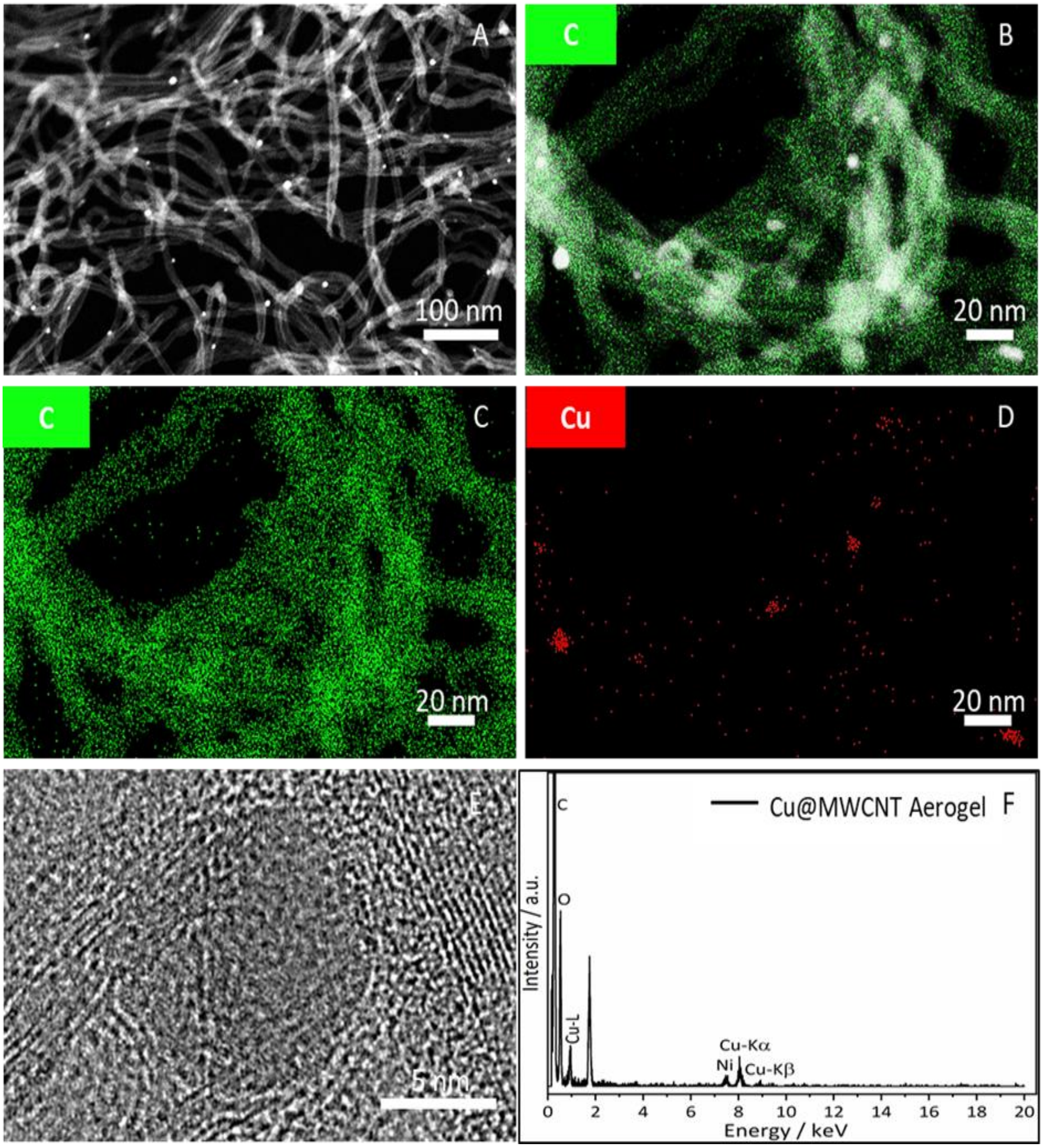

Figure S19. Electron microscopy and spectroscopy of Cu@MWCNT aerogels. A) HAADF-TEM of Cu-CuO core-shell NPs supported on MWCNT aerogel. B-D) EDX elemental mapping with carbon and copper tagged as green and red, respectively. E) Spherical Cu-CuO NP morphology. F) EDX map confirming the presence of Cu-CuO.

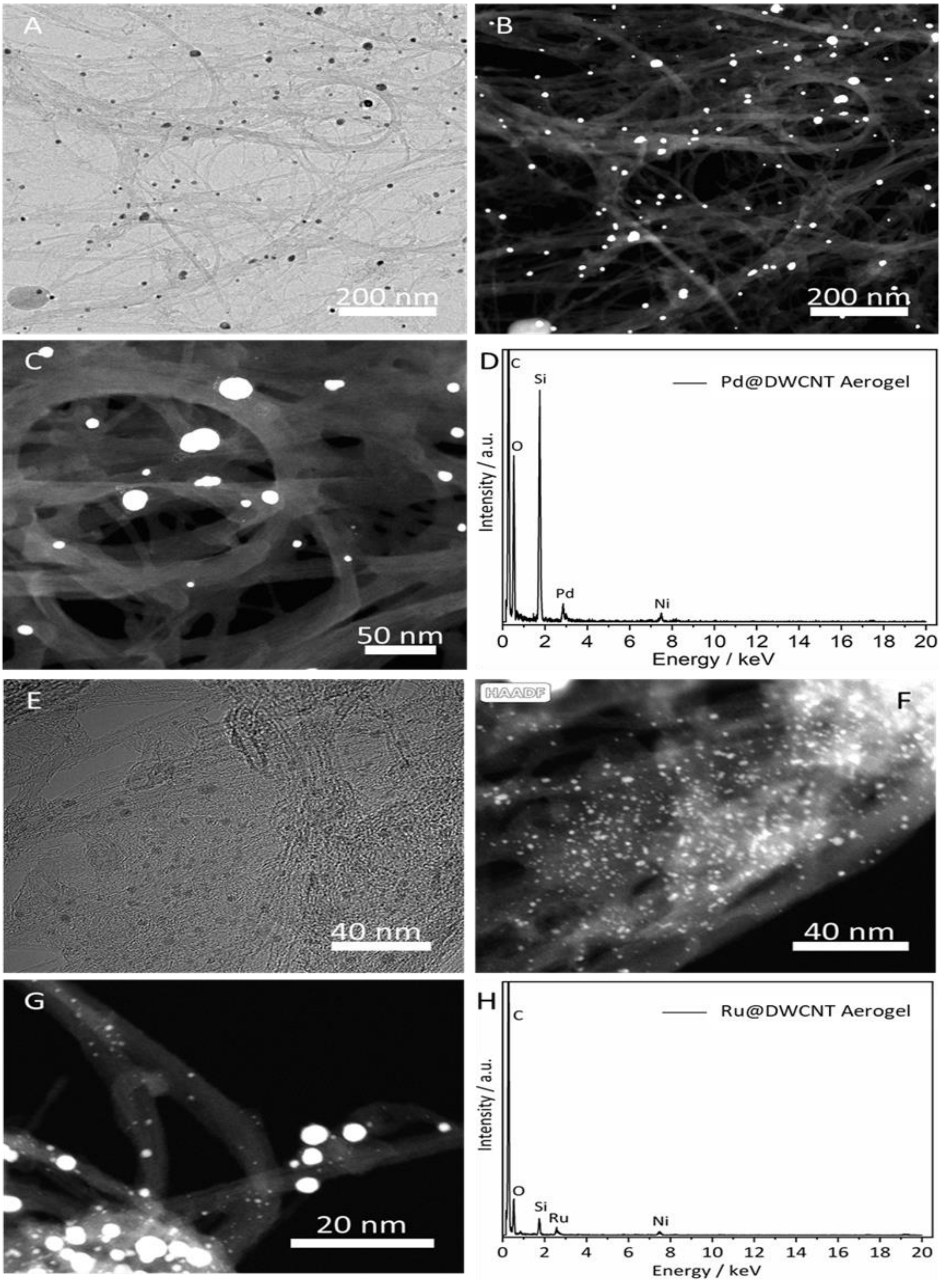

Figure S20. Electron microscopy and spectroscopy of Pd@DWCNT and Ru@DWCNT aerogels. (A) BF-TEM of Pd NPs uniformly supported on nanotubes. (B,C) HAADF-TEM of the supported Pd NPs. (D) EDX spectrum providing confirmation as to the elemental detection of palladium. In an identical layout; (E) BF-TEM of the supported Ru NPs. (F,G) HAADF-TEM of corresponding supported Ru NPs. (H) EDX spectrum showing the presence of Ru.

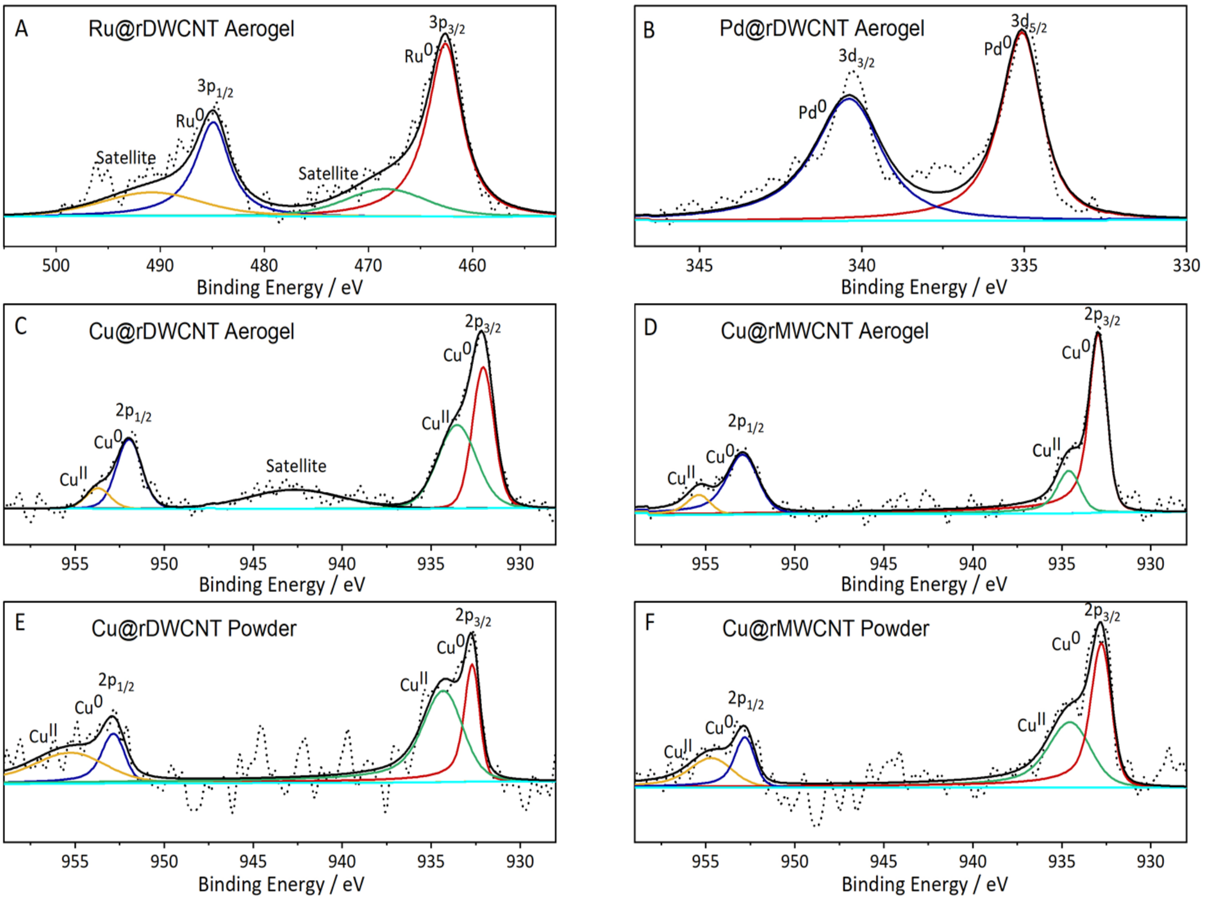

Figure S21. High-resolution XPS scans and fits for Cu-CuO, Pd and Ru NPs embedded in different aerogels and powders (DWCNTs and MWCNTs). (A) Ru@DWCNT aerogel with 3p peaks confirm the presence of Ru^0. (B) Pd@DWCNT aerogel with 3d peaks confirming the presence of Pd^0. (C,D) Cu@DWCNT and Cu@MWCNT aerogels both showing peaks for Cu^0 and Cu^{II}. (E,F) Corresponding powder analogues to the aerogels, also showing peaks for Cu^0 and Cu^{II}. Analysis of these spectra is found within the main text of the paper.

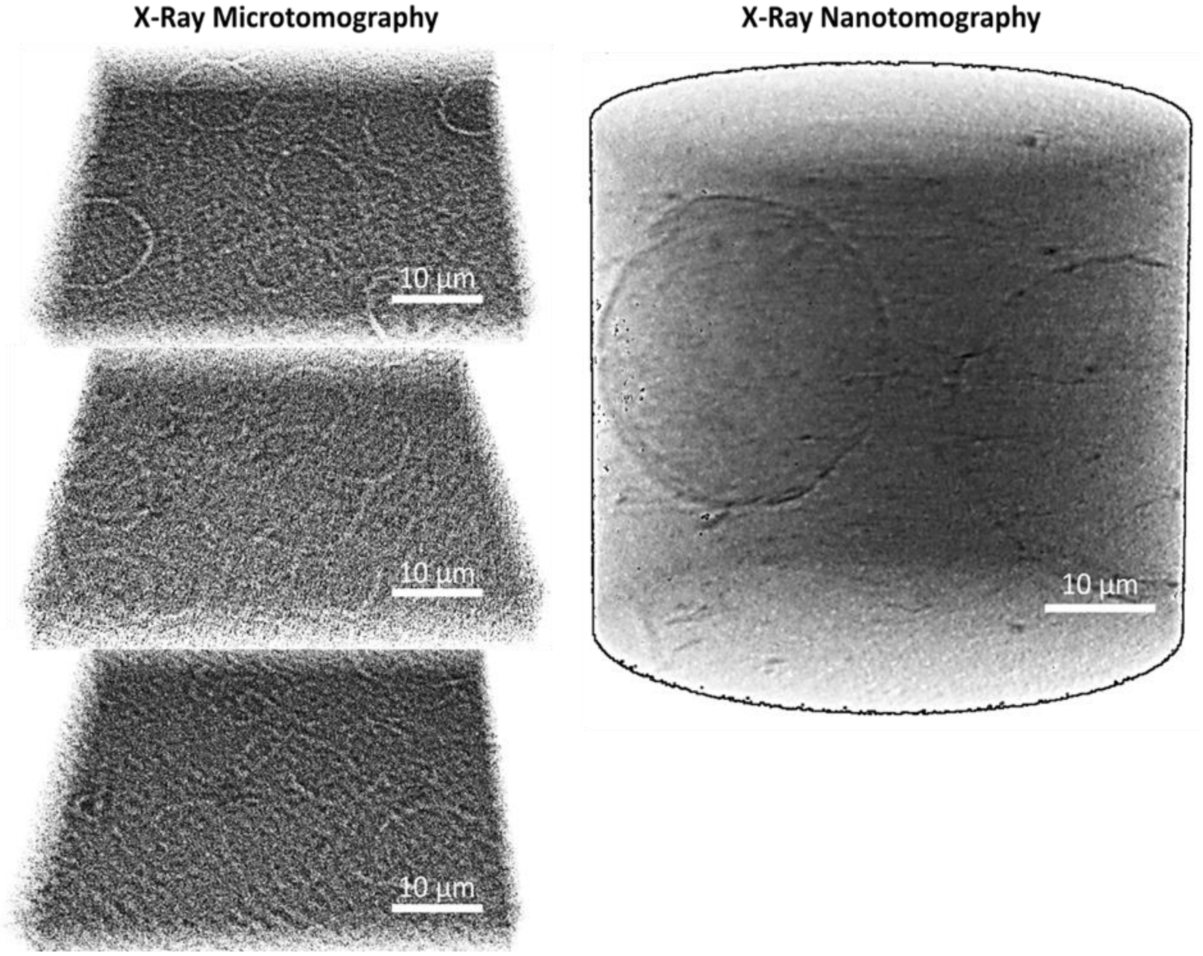

Figure S22. X-ray microtomography (left) and nanotomography (right) 3D reconstruction of orthoslices revealing the MWCNT microcages. X-ray microtomography shows multiple microcages bundled together and relatively uniform. X-ray nanotomography provides a high-resolution 3D reconstruction of two microcages without the presence of large Cu-CuO clusters reinforcing the SEM and TEM data of uniformly distributed monodisperse Cu-CuO core-shell NPs.

Catalytic Oxidative Amidation Reaction

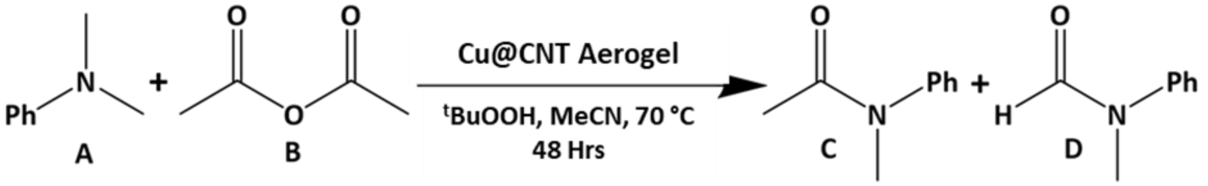

Scheme S1. Model amidation reaction between a tertiary amide and an acid anhydride. The reactants *N,N*-dimethylaniline (A) and acetic anhydride (B) react to form the desired product *N*-methyl-*N*-phenylacetamide product (C), with N-methyl-N-phenylformamide as main by-product (D). Cu-CuO decorated CNTs (Cu@CNTs) were employed as catalysts in this reaction at 70 °C in acetonitrile for 48 hrs with tertbutyl hydroperoxide as oxidising agent.

Oxidative Amidation Reaction

Reaction Pathway

Comparing Cu supported on CNT aerogels to Cu supported on zeolites in the same heterogeneous catalytic reaction, the most plausible reaction pathway proceeds via a single electron transfer from the N,N-dimethylaniline (limiting reactant) forming an aminium radical cation followed by a proton transfer resulting in an α-amino radical. A further single electron transfer transforms the α-amino radical into an iminium cation. This intermediate undergoes hydrolysis to N-hydroxymethylamine which reacts with the acetic anhydride to yield the desired N-methyl-N-phenylacetamide (desired product).[13] Although beyond the scope of this paper, it's likely that a redox process occurs whereby the TBHP oxidises the Cu species and the single electron transfer from the N,N-dimethylaniline reactant reduces the Cu species. The XPS results would indicate that the NP surface layer of Cu is involved possibly through a Cu^{I}/Cu^{II} couple (see XPS Figure S27).[14]

Conversion, Yield, TON and TOF Calculations

Yield calculations for reactant converted was based on H^1 NMR (400 MHz) integration of the limiting reactant N,N-dimethylaniline peak (6H singlet, 2.92 ppm) at 0 hr under the integrated value at a given time later (> 0 hr):

$$Overall\ Conversion\ (\%) = 100 - \left(\frac{Reactant\ Peak\ Area_{>0hr}}{Reactant\ Peak\ Area_{0hr}} x100\right)$$

Yield calculations for product formation was calculated in a similar way, with the numerator replaced with the integrated N-methyl-N-phenylacetamide peak (3H singlet, 2.23 ppm):

$$Product\ Formation\ (\%) = \left(\frac{Product\ Peak\ Area_{>0hr}}{Reactant\ Peak\ Area_{0hr}} x100\right)$$

Turnover number (TON), which considers the actual catalyst loading on the different nanotube supports determined from residual weight in the TGA (Table 2 and Figure S22) highlight the enhanced Cu-CuO activity on the nanotube aerogels. The turnover number (TON) is calculated from the ratio of product moles to Cu-CuO catalyst moles (TON):

$$TON = \frac{Moles_{Product\ Formed}}{Moles_{Cu\ Catalyst}}$$

The turnover frequency (TOF) is the TON adjusted for time (in this paper, TON and TOF calculations were determined at equilibrium after 48 hrs reaction duration):

$$TOF = \frac{TON}{X\ Hr}$$

Table S4. Summary of catalytic yields for all catalysts and reference materials. This table allows for relative comparison between different catalysts. The equilibrium (48 hrs) selectivity was calculated as ratio of reactant converted over product formed. Selectivity, TON and TOF values cement the hypothesis that the Cu-CuO core-shell NPs supported on CNT aerogels exhibit superior catalytic qualities compared to powder analogues and MWCNT-based catalysts.

Catalyst	Overall Conversion (%)	Desired Product Formation (%)	Selectivity (%)	TON	TOF (hr^{-1})
**Blank**	24	8	33	-	-
**MWCNT Aerogel**	17	4	24	-	-
**DWCNT Aerogel**	13	5	33	-	-
**Cu Nanopowder**	80	41	51	63	1
**Cu@MWCNT Powder**	86	50	58	86	2
**Cu@DWCNT Powder**	95	64	67	107	2
**Cu@MWCNT Aerogel**	92	57	62	70	1
**Cu@DWCNT Aerogel**	100	70	70	164	3

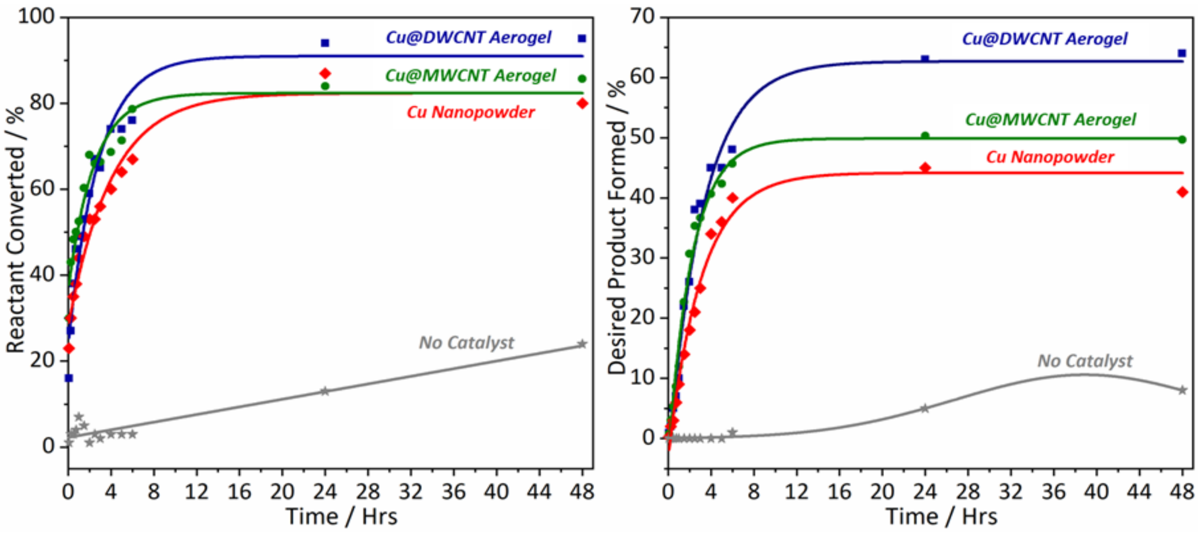

Figure S23. Limiting reactant (*N,N*-dimethylaniline) converted (left plot) and desired product (N-methyl-N-phenylacetamide) formation (right plot). Both plots show the superior catalytic activity of the Cu-CuO catalyst supported on DWCNTs in terms of both overall conversion and selectivity to the desired product. This trend is also observed for the performance of the aerogel catalysts, strongly supporting the notion that the DWCNTs function as better catalyst supports.

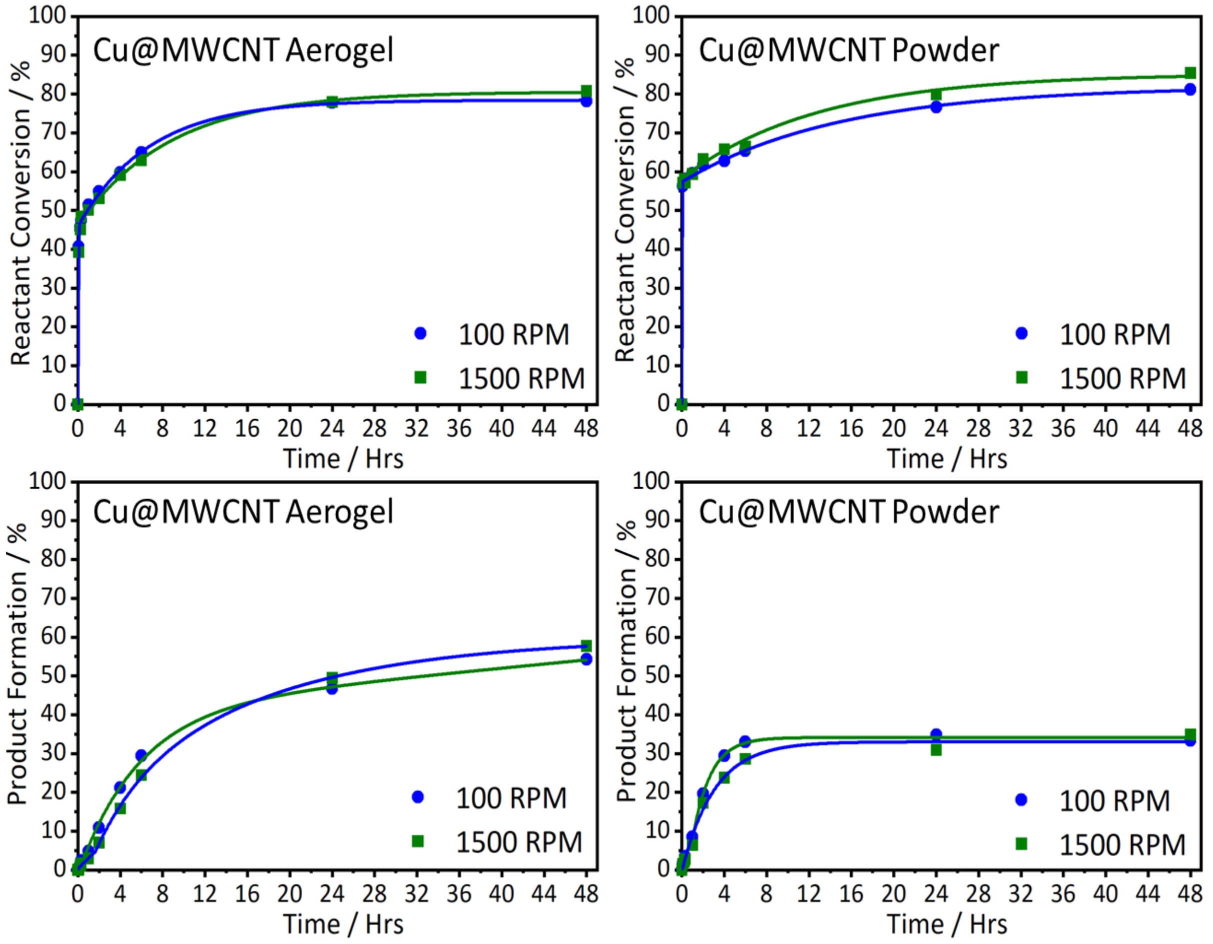

Figure S24. Impact of stirring rate on catalytic activity. Under the conditions utilised in this reaction, the stirring rate (between 100-1500 RPM) does not limit diffusion, enabling comparison between the catalytic performance of both aerogels and powders.

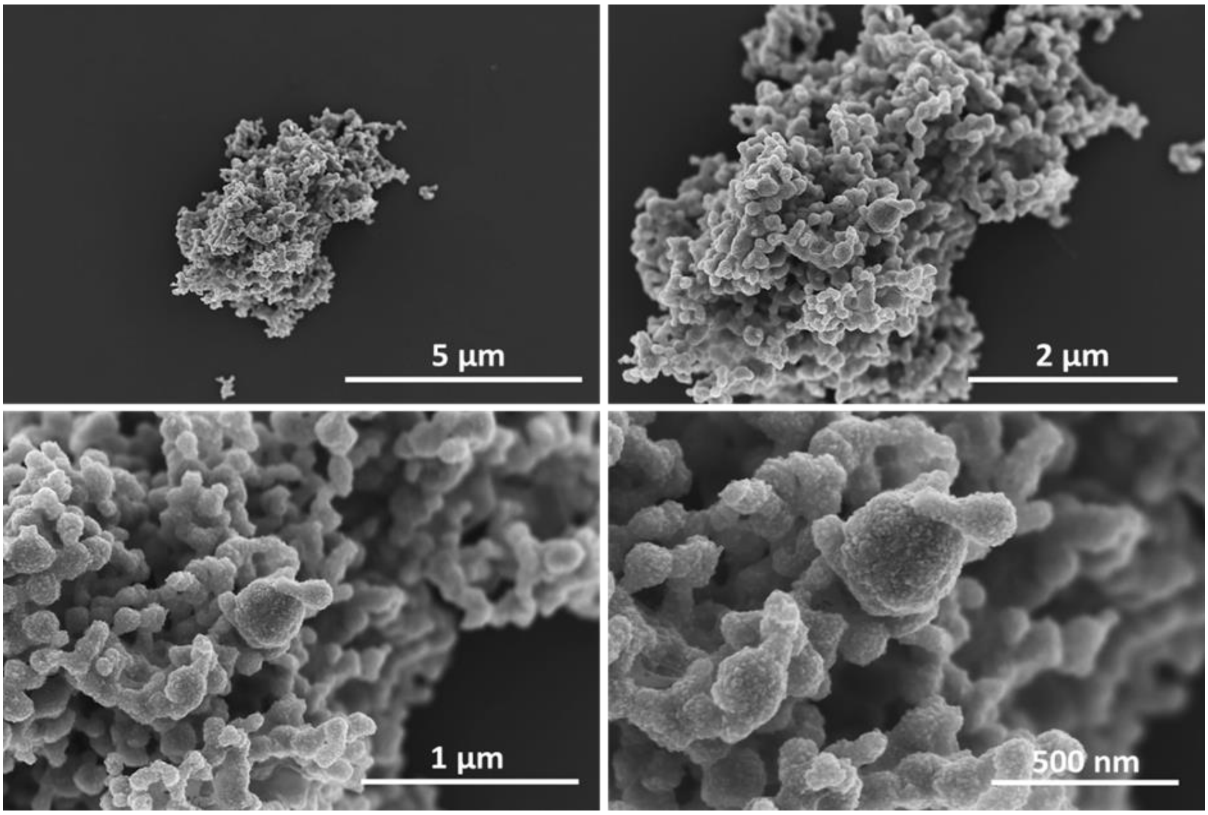

Figure S25. SEM images of Cu nanopowder. Small faceted Cu NPs (60-80 nm) appear aggregated into larger clusters. Cu nanopowder was used as a comparative catalytic material in the amidation reaction.

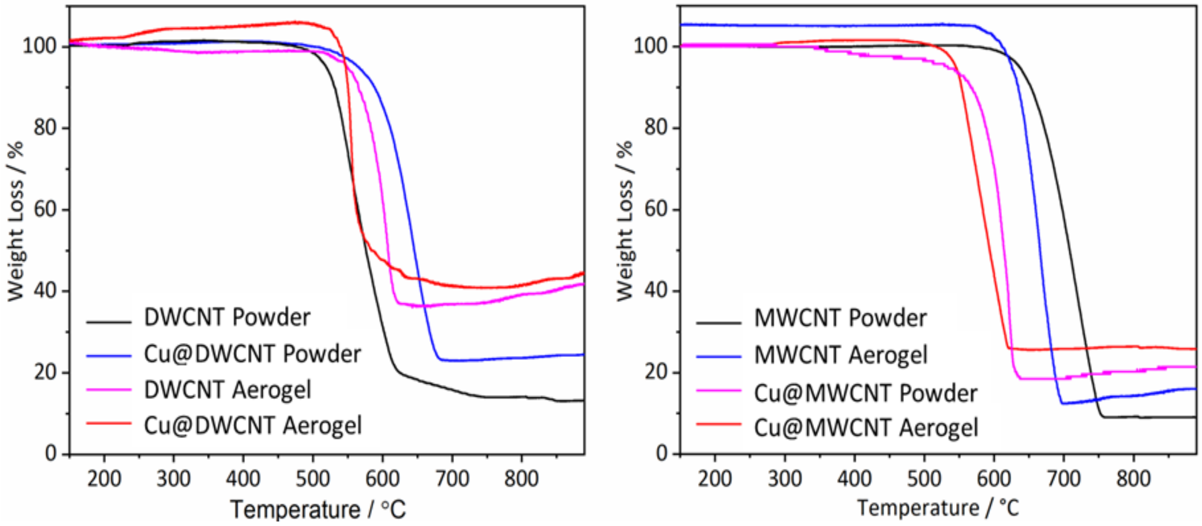

Figure S26. TGA of catalyst materials in reference to their undecorated counterparts. TGA residues were used to experimentally determine Cu-CuO catalyst loading in the decorated aerogel and powder materials.

Assessment of Catalytic Performance

Broader observations of catalytic performance of Cu@CNT aerogels reveal good catalytic activities when compared to other 3D porous structures, such as Cu-zeolites, employed in the same reaction.[15] In all reactions, the initial rate of conversion is extremely fast, with all Cu@MWCNT materials exhibiting a similar and high conversion of around 40% within the first 5 mins of the reaction (independent of stirring rate). These fast conversion kinetics make it challenging to accurately probe mass-transport effects during conversion in greater detail. In contrast, product formation kinetics are considerably slower. Here, we find again that initial rates for product formation are very similar for the two stirring rates probed, suggesting that there are no significant mass-transfer limitations. Potential reasons for this could be the relatively small reaction volume (5 cm^3 liquid reaction medium, compared to 0.2 cm^3 volume of the aerogel catalysts) and the pronounced macroporosity of the aerogel catalysts, that could mitigate against mass transfer issues.

However, this work reveals that our catalyst systems lie not only in improving reaction kinetics (especially as the commercial Cu nanopowder already shows good conversion activity at relatively mild reaction conditions), but much more in other features of the Cu@CNT aerogel systems. For example, the aerogel systems show improved product selectivity and reduced catalyst deactivation. These factors lead to a clear improvement in Cu utilisation efficiency in the aerogel-based catalyst systems, as evidenced by the tripling of the TON from Cu nanopowder (TON=63) to Cu@DWCNT aerogel (TON=164). The origin for the underlying differences in deactivation and selectivity are intriguing. Deactivation seems to be related to Cu-CuO oxidation during reaction. The changes in selectivity could be based on subtle differences in NP faceting, NP/CNT interactions or nanoscale confinement, imparted by different nanocarbon supports.

XPS analysis of the catalyst material prior to and after the reaction provide insight on product yield. The corresponding results indicate significant changes in the post-catalysis NPs which we hypothesis are an important reason behind the limited yields. Before the reaction, we observe the Cu $2p_{3/2}$ peak at 932.6 eV (attributed to the metallic Cu^0 core) with a pronounced shoulder at 934.6 eV (attributed to a Cu^{II} shell). However, after the reaction, the Cu $2p_{3/2}$ peak becomes more symmetrical with the peak maximum shifted to higher binding energies (934.6 eV) and the FWHM significantly increased. Similar observations are made for the Cu $2p_{1/2}$ peak. In addition, very prominent satellite features appear at 940.2 eV and 944.5 eV for the post-catalysis NPs. These XPS observations indicate that only Cu^{II} is present in the post catalysis NPs, while Cu^0 or Cu^I are absent in the surface layer. Similar observations are made for the powder analogues (despite the weak XPS signal).

These findings indicate that Cu-CuO core-shell NPs completely oxidise though over the course of the reaction and convert into pure CuO NPs, most likely due to oxidation by the strong oxidation agent (tert-butyl hydroperoxide) present in excess within the reaction. This conversion into pure CuO under the reaction conditions is likely linked to the deactivation of the catalyst at prolonged reaction times, although the underpinning deactivation pathway is less clear. Literature reports that catalytically active Cu-based NPs is typically attributed to Cu^I.[4] While Cu^I is not directly indicated in the XPS of the Cu-CuO core-shell NPs, its presence cannot be excluded (as it is difficult to distinguish Cu^0 and Cu^I in the XPS Cu 2p region).[16] It might also be possible that Cu^I is available through synproportionation reactions of Cu^0 and Cu^{II} in the original particles during reaction. In either case, complete oxidation to Cu^{II} would make Cu^I eventually unavailable in the reaction, leading to catalyst deactivation at prolonged reaction times. Future work could investigate the exact mechanism involved, opportunities to restore catalytic activity via simple thermal reductions as well as catalytic investigations of the noble metal NP (which are less prone to oxidation).

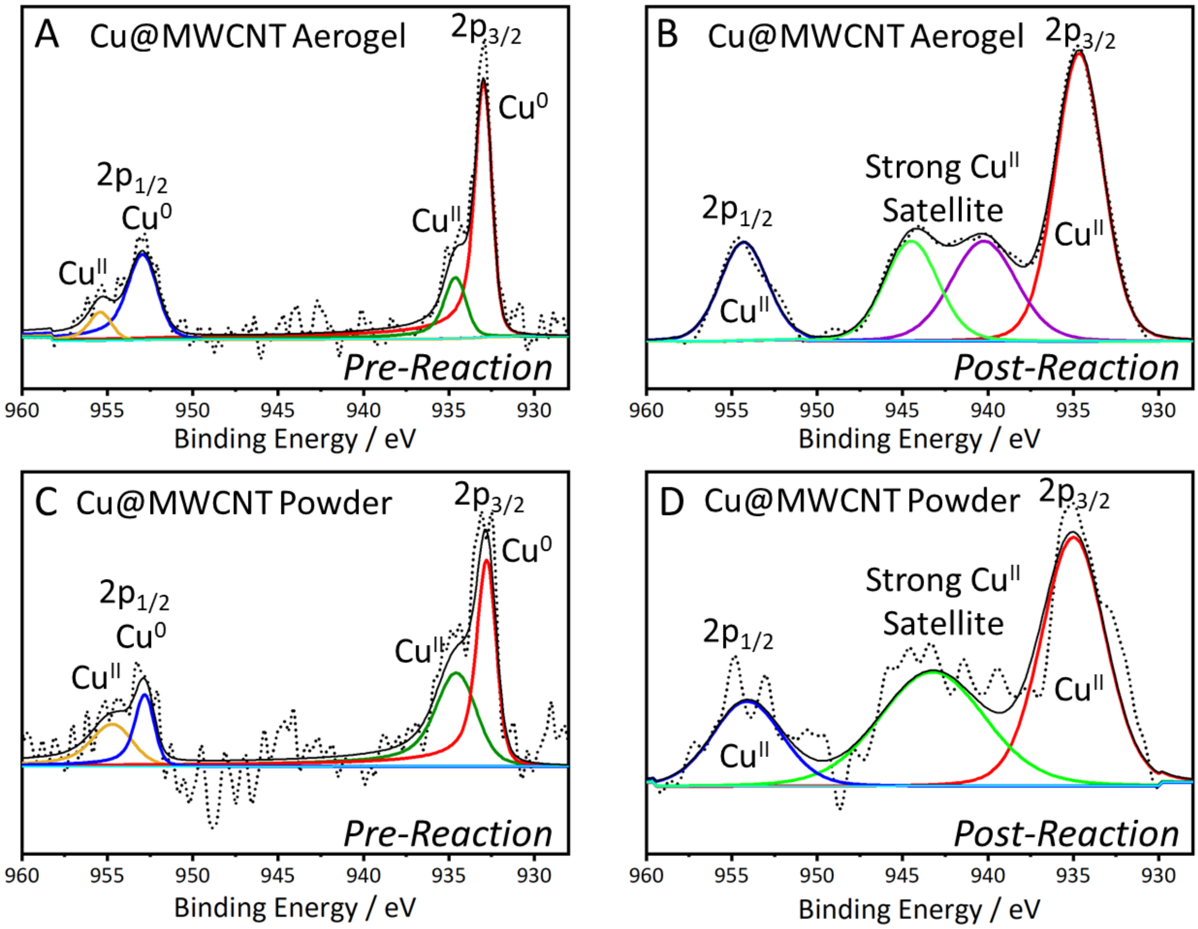

Figure S27. XPS analysis of the Cu 2p spectra before and after exposure to the oxidative amidation reaction. The deconvoluted XPS peaks evidence the oxidative transformation of a Cu^0-Cu^{II} catalytic surface into purely Cu^{II} providing insight on potential deactivation processes.

References

[1] Y. Q. Li, T. Yu, T. Y. Yang, L. X. Zheng, K. Liao, *Adv. Mater.* **2012**, *24*, 3426.

[2] J. N. Coleman, M. Cadek, R. Blake, V. Nicolosi, K. P. Ryan, C. Belton, A. Fonseca, J. B. Nagy, Y. K. Gun'ko, W. J. Blau, *Adv. Funct. Mater.* **2004**, *14*, 791.

[3] S. Barg, F. M. Perez, N. Ni, P. do Vale Pereira, R. C. Maher, E. Garcia-Tuñon, S. Eslava, S. Agnoli, C. Mattevi, E. Saiz, *Nat. Commun.* **2014**, *5*, 4328.

[4] P. Papakonstantinou, H. Murphy, J. A. Mclaughlin, N. M. D. Brown, *Carbon* **2005**, *43*, 153.

[5] R. Larciprete, S. Gardonio, L. Petaccia, S. Lizzit, *Carbon* **2009**, *47*, 2579.

[6] K. A. Worsley, I. Kalinina, E. Bekyarova, R. C. Haddon, *J. Am. Chem. Soc.* **2009**, *131*, 18153.

[7] H. Yu, Y. Jin, F. Peng, H. Wang, J. Yang, *J. Phys. Chem. B* **2008**, *112*, 6758.

[8] T. Bortolamiol, P. Lukanov, A. Galibert, B. Soula, P. Lonchambon, L. Datas, E. Flahaut, *Carbon* **2014**, *78*, 79.

[9] F. Tuinstra, J. L. Koenig, *J. Chem. Phys.* **1970**, *53*, 1126.

[10] A. C. Ferrari, J. C. Meyer, V. Scardaci, C. Casiraghi, M. Lazzeri, F. Mauri, S. Piscanec, D. Jiang, *Phys. Rev. Lett.* **2006**, *97*, 187401.

[11] A. C. Ferrari, J. Robertson, *Phys. Rev. B* **2000**, *61*, 14095.

[12] Y. Yao, Z. Huang, P. Xie, S. D. Lacey, R. J. Jacob, H. Xie, F. Chen, A. Nie, T. Pu, M. Rehwoldt, D. Yu, M. R. Zachariah, C. Wang, R. Shahbazian-Yassar, J. Li, L. Hu, *Science* **2018**, *359*, 1489.

[13] Y. Li, L. Ma, F. Jia, Z. Li, *J. Org. Chem.* **2013**, *78*, 5638.

[14] F. Alonso, A. Arroyo, I. Martín-garcía, Y. Moglie, *Adv. Synth. Catal.* **2015**, *357*, 3549.

[15] Y. Moglie, E. Buxaderas, A. Mancini, F. Alonso, G. Radivoy, *ChemCatChem* **2019**, *11*, 1487.

[16] C. K. Wu, M. Yin, S. O'Brien, J. T. Koberstein, *Chem. Mater.* **2006**, *18*, 6054.
